# Pathway-based Screening Strategy for Multitarget Inhibitors of Diverse Proteins in Metabolic Pathways

**DOI:** 10.1371/journal.pcbi.1003127

**Published:** 2013-07-04

**Authors:** Kai-Cheng Hsu, Wen-Chi Cheng, Yen-Fu Chen, Wen-Ching Wang, Jinn-Moon Yang

**Affiliations:** 1 Institute of Bioinformatics and Systems Biology, National Chiao Tung University, Hsinchu, Taiwan; 2 Institute of Molecular and Cellular Biology & Department of Life Sciences, National Tsing Hua University, Hsinchu, Taiwan; 3 Institute of Molecular Biology, Academia Sinica, Taipei, Taiwan; 4 Department of Biological Science and Technology, National Chiao Tung University, Hsinchu, Taiwan; 5 Center for Bioinformatics Research, National Chiao Tung University, Hsinchu, Taiwan; The Pennsylvania State University, United States of America

## Abstract

Many virtual screening methods have been developed for identifying single-target inhibitors based on the strategy of “one–disease, one–target, one–drug”. The hit rates of these methods are often low because they cannot capture the features that play key roles in the biological functions of the target protein. Furthermore, single-target inhibitors are often susceptible to drug resistance and are ineffective for complex diseases such as cancers. Therefore, a new strategy is required for enriching the hit rate and identifying multitarget inhibitors. To address these issues, we propose the pathway-based screening strategy (called PathSiMMap) to derive binding mechanisms for increasing the hit rate and discovering multitarget inhibitors using site-moiety maps. This strategy simultaneously screens multiple target proteins in the same pathway; these proteins bind intermediates with common substructures. These proteins possess similar conserved binding environments (pathway anchors) when the product of one protein is the substrate of the next protein in the pathway despite their low sequence identity and structure similarity. We successfully discovered two multitarget inhibitors with IC_50_ of <10 µM for shikimate dehydrogenase and shikimate kinase in the shikimate pathway of *Helicobacter pylori*. Furthermore, we found two selective inhibitors (IC_50_ of <10 µM) for shikimate dehydrogenase using the specific anchors derived by our method. Our experimental results reveal that this strategy can enhance the hit rates and the pathway anchors are highly conserved and important for biological functions. We believe that our strategy provides a great value for elucidating protein binding mechanisms and discovering multitarget inhibitors.

## Author Summary

Many drug development strategies focus on designing inhibitors for single targets. These inhibitors often lose potency owing to mutations in the protein binding sites and are ineffective for complex diseases. Multitarget inhibitors can decrease probability of drug resistance and enhance the therapeutic efficiency; however, identifying them is still a challenge because targets often have low sequence and structure similarities in their binding sites. Here we propose a pathway-based screening strategy that simultaneously screens proteins in a metabolic pathway for discovering multitarget inhibitors. Because these proteins interact with similar metabolites and modify them step-by-step, the proteins share similarities in binding sites. We developed pathway site-moiety maps that present the conserved binding environments of the proteins without relying on the sequence or structure alignment. Compounds that bind these conserved binding environments are often multitarget inhibitors. We applied this strategy to the shikimate pathway of *Helicobacter pylori*, and discovered two multitarget inhibitors (IC_50_<10 µM) for shikimate dehydrogenase and shikimate kinase. In addition, we found two selective inhibitors based on specific binding environments for shikimate dehydrogenase. Thus the pathway-based screening strategy is useful for identifying multitarget inhibitors and elucidating protein-ligand binding mechanisms and has the potential to be applied to human diseases.

## Introduction

The concept of “one–disease, one–target, one–drug” has dominated drug development strategy for decades [1], [2]. Based on this strategy, many virtual screening methods have been developed and applied successfully for identifying specific inhibitors of a single target [3]–[5]. However, the hit rates of these screening methods are often low because they generally cannot identify the key features from a single protein for understanding biological functions or determining inhibitor activities. In addition, single-target inhibitors often lose their potency owing to a single residue mutation in the target binding sites, resulting in drug resistance. For instance, some influenza strains containing a single residue mutation are resistant to the drug oseltamivir [6]. Another well-known example is human immunodeficiency virus type 1, which has a high mutation rate and rapidly develops resistance to drugs [7]. Furthermore, the single-target inhibitors are often therapeutically inefficient for complex diseases that were caused by multiple targets (such as cancers). Therefore, an emerging strategy for drug discovery is to enrich the hit rate and identify multitarget inhibitors, decreasing the probability of drug resistance and enhancing therapeutic efficiency by inhibiting multiple targets.

Some proteins share similarities in physicochemical properties and shapes of their localized binding sites despite low sequence or low overall structural similarities. For example, the proteins in a metabolic pathway contain conserved binding environments where the product of one enzyme is the substrate of the next enzyme in a series of catalytic reactions. Using this property, it is possible to design a multitarget inhibitor to simultaneously inhibit multiple proteins in a disease pathway to increase the therapeutic effectiveness against the disease. Recently, the concept of polypharmacology has been proposed for drug design, which deals with drugs that bind multiple target proteins [8]–[10]. However, designing these inhibitors is a challenging task because the proteins in a pathway often lack structural and sequence homology [11], [12]. Therefore, a new strategy for extracting conserved binding environments from these proteins is needed for the discovery of multitarget inhibitors.

To address these issues, we propose a new strategy, called pathway-based screening by using pathway site-moiety maps (PathSiMMaps). The strategy is an extension of our previous studies, which described a site-moiety map of a protein and core site-moiety maps of orthologous proteins [13], [14]. The main concept of this strategy is the simultaneous screening of multiple target proteins in the same metabolic pathway that interact with compounds sharing similar common substructures. Our previous studies showed that a site-moiety map can identify the moiety preferences and the physico-chemical properties of a binding site for elucidating binding mechanisms [13], [14]. A site-moiety map contains several anchors, and an anchor has three essential elements: (1) conserved interacting residues of a binding pocket (*i.e.*, a part of the binding site); (2) moiety preference of the binding pocket; and (3) the type of interaction between the moieties and the binding pocket. Here we have extensively enhanced and modified our previous works to develop PathSiMMaps of multiple proteins with low sequence and structural similarities using an anchor-based alignment method. PathSiMMaps represents the conserved binding environments (*i.e.*, conserved anchors, called pathway anchors) of multiple proteins in a pathway. Pathway anchors often play key roles in the series of catalytic reactions, and therefore can be used for the discovery of multitarget inhibitors and to increase the hit rate.

The major enhancements in PathSiMMaps developed in the present study compared with the core site-moiety maps developed in our previous study are as follows. The PathSiMMaps are designed to identify multitarget inhibitors for multiple proteins lacking structural similarity and sequence homology. In contrast, the core site-moiety maps were designed for orthologous proteins, which often have the same functions and similar structures in their binding sites. An anchor-based alignment method was developed to identify pathway anchors without relying on sequence or structure alignments. The PathSiMMaps are also designed to find specific anchors and selective inhibitors for a specific protein. Furthermore, we developed a PathSiMMap-based scoring method to enrich the hit rate.

We have applied the pathway-based screening strategy to identify pathway anchors and multitarget inhibitors of shikimate dehydrogenase (SDH) and shikimate kinase (SK) in the shikimate pathway of *Helicobacter pylori* (*H. pylori*), which causes peptic ulcer disease [15], [16]. The shikimate pathway consisting of seven proteins is an attractive target pathway for drug development because it is absent in humans [17]. SDH and SK are among the seven proteins in the pathway. We first identified four pathway anchors of SDH and SK despite their low sequence identity (8.3%) and structure similarity (RMSD is 4.8 Å). Based on these pathway anchors, two multitarget inhibitors were successfully discovered for SDH and SK with IC_50_ of <10 µM. Experimental results show that pathway anchors and their residues are highly conserved and play important roles for studying biological functions and designing multiple-target inhibitors. Our screening strategy significantly enriches the hit rate based on multiple targets. These experimental results reveal that the pathway-based screening strategy can find pathway anchors and multitarget inhibitors of structurally dissimilar proteins. We believe that our strategy is useful for studying protein-inhibitor binding mechanisms and discovering multitarget inhibitors for human complex diseases.

## Results/Discussion

### Overview of pathway-based screening strategy

**Figure 1:**
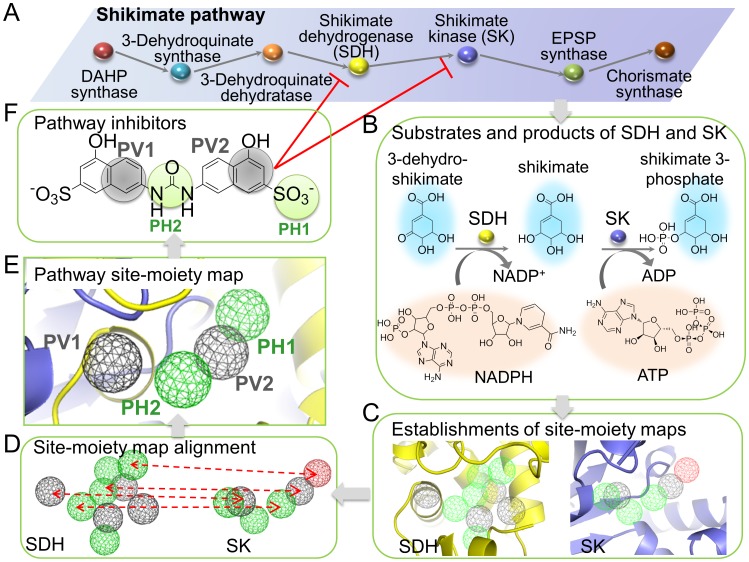
Overview of pathway-based screening strategy for identifying conserved binding environments and multitarget inhibitors of multiple proteins. (A) The proteins in the shikimate pathway. (B) Chemical reactions of shikimate dehydrogenase (SDH) and shikimate kinase (SK). SDH converts 3-dehydroshikimate into shikimate using NAPDH and then SK converts shikimate into shikimate 3-phosphate by ATP. These three compounds share the same substructure (blue region). (C) Establishment of site-moiety maps of SDH and SK. A site-moiety map represents the binding environments of a protein binding site by anchors. (D) The site-moiety map alignment of SDH and SK for identifying the conserved binding environments (pathway anchors). (E) Pathway anchors of SDH and SK. Hydrogen-bonding and van der Waals anchors are colored in green and gray, respectively. (F) Pathway inhibitors identified by the pathway anchors.

The concept of the pathway-based screening strategy is to simultaneously screen multiple proteins in a pathway and extract conserved binding environments of these proteins to discover multitarget inhibitors (Fig. 1). The screening strategy relies on the following criteria: (1) the proteins are in the same pathway; (2) the proteins catalyze similar ligands with common substructures; and (3) the site-moiety maps of these proteins share comparable pathway anchors. The strategy can work efficiently when proteins in the same pathway perform a series of catalytic reactions to yield a product compound. The intermediates of these proteins often share common substructures and their binding sites may share similar physicochemical properties and shapes.

Seven proteins in the shikimate pathway catalyze several metabolites with similar substructures to synthesize chorismate [18] (Figs. 1A, 1B, and S1). The similarities of the substrates, products and cofactors were represented by MACCS-Tanimoto values obtained from OpenBabel (http://openbabel.org/wiki/Main_Page) (Figs. S1B and S1C). The similarity matrix showed that the substrates/products generally share similarities (average Tanimoto value: 0.61). After 3-Dehydroquinate synthase converts DAHP to DHQ by NAD+, the downstream substrates/products (3-dehydroshikimate, shikimate, shikimate-3-phosphate, EPSP, and chorismate) of the protein have relatively similarities (Tanimoto value: 0.75) because these substrates/products share similar scaffolds (blue part in Fig. S1A). The cofactors in this pathway also have similar scaffolds (Fig. S1C).

In this study, we selected two proteins as the test screening targets: SDH and SK. These proteins are the fourth and fifth enzymes, respectively, in the shikimate pathway. SDH converts 3-dehydroshikimate into shikimate using NADPH as a cofactor [18] (Fig. 1B). Then, SK converts shikimate into shikimate 3-phosphate by another cofactor, ATP [19]. The major scaffolds (blue part) of 3-dehydroshikimate, shikimate, and shikimate 3-phosphate are the same (blue part in Fig. 1B), which implies that SDH and SK have conserved binding environments for recognizing this common scaffold.

We used the anchors of site-moiety maps to describe the binding environments of protein binding sites. The anchors have three interaction types: electrostatic (E), hydrogen-bonding (H), and van der Waals (V) interactions. First, we docked 302,909 compounds collected from public compound databases to binding sites of SDH and SK using our in-house docking tool, GEMDOCK [20]. Our previous studies revealed that GEMDOCK has similar performance to other docking methods such as DOCK [21], FlexX [22], and GOLD [20], [23], [24]. Furthermore, we have successfully used GEMDOCK to identify novel inhibitors and binding sites for several targets [25]–[27]. Subsequently, the site-moiety maps of SDH and SK were established by statistical analysis of the top 6,000 docked compounds (approximately 2% of the 302,909 compounds) (Fig. 1C). We then developed an anchor-based alignment method to find pathway anchors that are conserved in SDH and SK, for constructing the PathSiMMaps (Fig. 1D). The pathway anchors of the PathSiMMaps reflect conserved interactions between binding pockets with specific physico-chemical properties and their preferred functional groups, all of which are essential for pathway functions (Fig. 1E). Finally, the compounds that simultaneously matched pathway anchors of multiple targets were selected for the bioassay (Fig. 1F).

### Site-moiety maps of SDH and SK

The site-moiety map of SDH consisted of five H anchors (H1, H2, H3, H4, and H5) and four V anchors (V1, V2, V3, and V4) (Figs. 1C and S2). For each anchor, several residues comprising a binding pocket with specific physicochemical properties, moiety compositions, and interaction type were identified from the top-ranked compounds. The H1 anchor (Fig. S2C), consisting of three residues (T65, K69, and D105), prefers polar moieties such as carbonyl, amide, and nitro groups. The hydroxyl moiety of shikimate participates in the dehydrogenase reaction (Fig. 1B) and forms hydrogen bonds with the three residues of the H1 anchor (Fig. S2B). The two residues (K69 and D105) of this anchor are highly conserved and are responsible for transferring a hydride ion between NADPH and shikimate in SDH of *Thermus thermophilus* [28]. Three residues (H15, T65, and Y210), constituting the H2 anchor, form hydrogen bonds with amide, carbonyl, sulfonate, amide, and carboxylic acid groups of the top-ranked compounds. The H4 (S129, A179, and T180) and H5 (K69 and S129) anchors interact with NADPH and are composed of two polar binding pockets (Fig. S2B). The major interacting moieties of the H4 anchor are carboxylic acid amide, ketone, ether, and hydrazine derivatives. The H5 anchor favors carboxylic acid amide, ketone, sulfonate, and carboxylic acid groups. The H3 anchor, which is spatially distant from the shikimate and NADPH binding sites, consists of three residues (T180, D207, and L208), revealing an additional binding pocket for designing inhibitors. This binding pocket often forms interactions with nitro, ether, sulfonate, and ketone moieties.

Ring moieties are the major moiety types of the V1, V2, V3, and V4 anchors of SDH. Among the 6000 top-ranked compounds, the aromatic moieties of 1,879; 886; 745; and 1,454 compounds form van der Waals contacts with the residues of the V1, V2, V3, and V4 anchors, respectively. The number of compounds (3,509 of 6,000 compounds) interacting with the V1 anchor, formed by three hydrophobic residues (L66, L208, and A209), is higher than those for the other V anchors. Aromatic ring, phenol, alkene, and oxohetarene moieties are the major compositions of the V1 anchor. The ribose of the cofactor NADPH is located in the V1 anchor (Fig. S2B), suggesting the importance of the V1 anchor for maintaining the function of the protein. The V2 anchor constituted by three residues (L208, Y210, and Q237) forms van der Waals interactions with 1,933 docked compounds by bulky moieties such as aromatic and heterocyclic moieties. Furthermore, this anchor occupies the position of the pyridine ring of NADPH (Fig. S2B). The V4 and the V3 anchors are situated in the groove and close to the entrance of the NADPH binding site, respectively. The residues (L66, G127, and G128) of the V4 anchors often interact with aromatic ring, phenol, alkene, and oxohetarene moieties. The three residues (L184, A209, and Y210) comprise the V3 anchor, and their preferred moieties are aromatic ring, alkene, phenol, and oxohetarene moieties. These nine anchors (five H and four V anchors) describe binding environments that can be used to design SDH inhibitors that block the binding of shikimate or NADPH.

We have previously described the site-moiety map of SK [14]. Three anchors (E1, V2, and H3) are located at the shikimate binding site (Fig. S3). The E1 anchor pocket consists of two positively charged residues (R57 and R132) which are essential for shikimate binding [29]. The anchor prefers negatively charged moieties such as carboxyl, sulfonate, and phosphate groups. The V2 anchor residues (D33, F48, G80, and G81) form van der Waals interactions with the ring of shikimate (Fig. S3B). This pocket often interacts with aromatic rings, carboxylic acid amidine, oxohetarene, and alkene moieties. The polar pocket of the H3 anchor consists of three residues (K14, D33, and G80) which often form hydrogen-bonding interactions with polar moieties (carboxylic acid amide, ketone, sulfonate, and ether) of the docked compounds. The H1, H2, and V1 anchors are situated at the ATP site. The H1 (G11, S12, G13, K14, and S15) and H2 (S15, D31, and D33) anchors are involved in the Walker A motif (K14 and S15) and a DT/SD motif (D31 and D33), respectively, and bind the phosphate groups of ATP [29]. The two anchors favor similar polar moieties, such as carboxylic acid amide, ketone, and sulfonate. The V1 anchor (M10, G11, S12, G13, K14, and S15) is situated between the H1 anchor and the H2 anchors, and its frequently interacting moieties are aromatic groups, oxohetarene, phenols, heterocyclic groups, and alkenes.

### Pathway site-moiety map of SDH and SK

**Figure 2:**
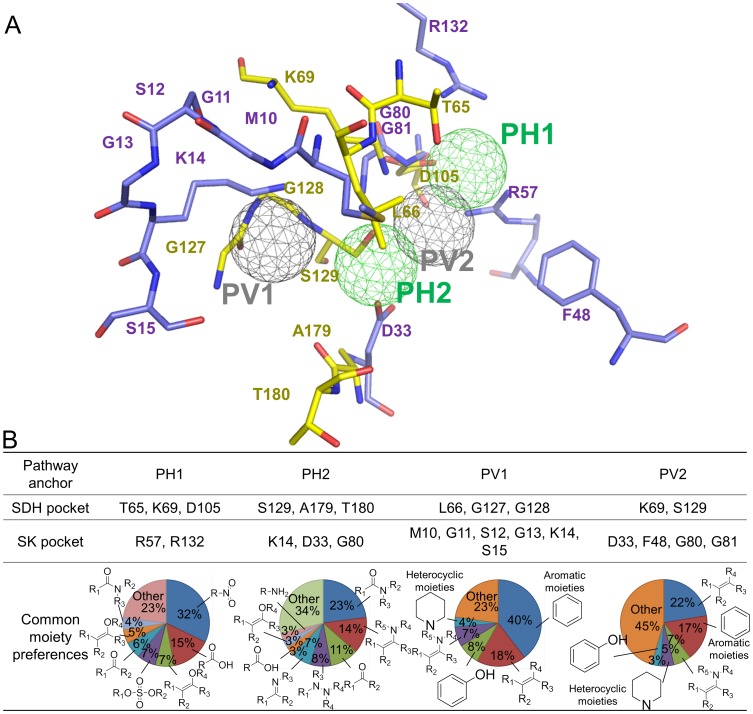
Pathway site-moiety map of SDH (yellow) and SK (purple). (A) Superimposed binding sites of SDH and SK by the anchor-based alignment method. Four pathway anchors were identified despite low sequence and structure similarities of SDH and SK. Hydrogen-bonding and van der Waals anchors are colored in green and gray, respectively. (B) Common moiety preferences of four pathway anchors.

**Figure 3:**
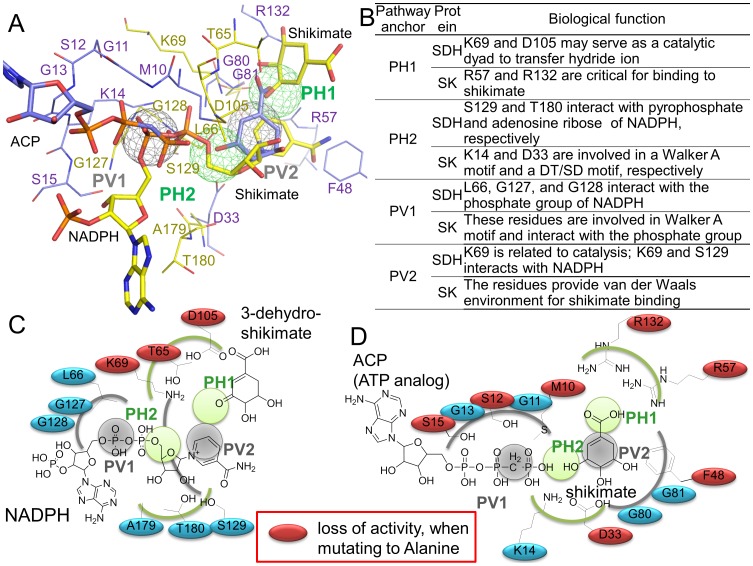
Relationship between the pathway anchors and biological functions. (A) Ligands of SDH and SK in the pathway site-moiety map. The ligands include shikimate and NADPH of SDH (PDB code 3PHI), and shikimate and ACP (ATP analog) of SK (PDB code 1ZUH [39], a shikimate kinase structure of *Mycobacterium tuberculosis*). (B) Pathway anchors for biological functions. Diagrams of the ligands and the pathway anchors for (C) SDH and (D) SK. Residues are colored in red if their mutations lead to loss of enzyme activity [14], [19], [30], [31].

SDH and SK have four pathway anchors identified by the anchor-based alignment method despite their low sequence and structure similarity (Figs. 2 and 3). The pathway hydrogen-bonding anchor 1 (PH1) was derived from alignment of the H1 anchor of SDH and the E1 anchor of SK. The interaction type of the PH1 anchor was assigned as the hydrogen-bonding type because the preferred moieties of the E1 anchor are able to participate in hydrogen bonding. The pathway hydrogen-bonding anchor 2 (PH2) was derived from the alignment of the H4 anchor of SDH and the H3 anchor of SK. The pathway van der Waals anchor 1 (PV1) was derived from the alignment of the V4 anchor of SDH and the V1 anchor of SK. The pathway van der Waals anchor (PV2) was derived from alignment of the H5 anchor of SDH and the spatially close V2 anchor of SK.

The PH1 anchor consists of residues T65, K69, and D105 for SDH and R57 and R132 for SK (Fig. 2). The PH1 anchor prefers polar moieties such as nitro and carboxylic acid groups and is involved in the dehydrogenase reaction for SDH and the binding of shikimate [29] (Figs. 2B and 3). Interestingly, the shikimates of SDH and SK consistently occupy the location of the PH1 anchor. This result indicates that the PH1 anchor is essential for catalysis and substrate binding of these two proteins in the shikimate pathway. For SDH, the residues (S129, A179, and T180) of the PH2 anchor interact with NADPH; similarly, the SK residues (K14, D33, and G80) of PH2 are involved in the Walker A motif and DT/SD motif, both of which are involved in shikimate and ATP binding to SK [19] (Fig. 3). This suggests that the PH2 anchor is involved in shikimate binding and the binding of cofactors such as NADPH of SDH and ATP of SK.

The interaction residues of the PV1 anchor (L66, G127, and G128 in SDH; M10, G11, S12, G13, K14, and S15 in SK) constitute a binding pocket that frequently yields van der Waals interactions with compound moieties (Fig. 2B). The major moieties of the PV1 anchor are aromatic ring (40%), alkene (18%), and phenol (8%). The high preference of the aromatic ring may derive from the long side chains of the residues (L66 in SDH; M10 in SK), which can form stable van der Waals interactions with the aromatic rings of the compounds. For the PV1 anchor of SDH, the anchor residues (L66, G127, and G128) interact with the phosphate group of NADPH through van der Waals interactions. In addition, the residue L66 yields van der Waals interactions with the adenosine ribose of NADPH, which may stabilize NADPH binding. Similarly, the anchor residues (M10, G11, S12, G13, K14, and S15) of the SK PV1 anchor surround the phosphate groups of ATP and provide van der Waals interactions with ATP. These observations showed that the PV1 anchor plays an important role in interacting and transferring the phosphate groups of ATP (SK) and NADPH (SDH) during catalytic reactions, despite the different functions of SDH and SK (Fig. 3).

For the PV2 anchor, the side chains of its interaction residues (K69 and S129 in SDH; D33, F48, G80, and G81 in SK) provide van der Waals contacts with alkene (22%), aromatic ring (17%), enamine (7%), and heterocyclic moieties (5%) (Fig. 2B). The aromatic ring composition of the PV2 anchor is lower than that of the PV1 anchor, which may have resulted from the less compact binding environment of the PV2 anchor comprising a relatively small residue number. For SDH, the van der Waals interactions are formed between the residues (K69 and S129) of the PV2 anchor and the pyridine ring of NADPH (Fig. 3). Moreover, the residue K69 is a catalytic residue for the dehydrogenase reaction based on the SDH structure of *Thermus thermophilus* [28]. The SK PV2 anchor is located at the shikimate binding site, and its residues (D33 and F48) make van der Waals interactions with the cyclohexene group of shikimate. D33A or F48A mutations result in a loss of SK activity [14], revealing the anchor is essential for the shikimate binding. Although SDH and SK have different residue compositions in their PV2 anchors, these residues interact with similar ring moieties (e.g., cyclohexene of shikimate and the pyridine ring of NADPH) during their catalytic processes.

### Site-directed mutagenesis and new multitarget inhibitors

We evaluated the pathway anchors by site-directed mutagenesis. A site-directed mutagenesis study on SDH of *Escherichia coli* showed that it lost substrate-binding activity when the residues were mutated at positions 67, 92, and 107 (T65, J69, and D105, respectively in SDH of *H. pylori*) [30]. Our previous study also showed that mutations in the pathway anchor residues (M10, S12, S15, D33, F48, R57, and R132 in SK) reduced the activity of shikimate kinase [14], [31]. These results suggest that the pathway anchors are essential for catalytic reactions and that the mutations on the pathway anchor resides often decrease enzyme activities of SDH and SK (Figs. 3C and 3D).

**Figure 4:**
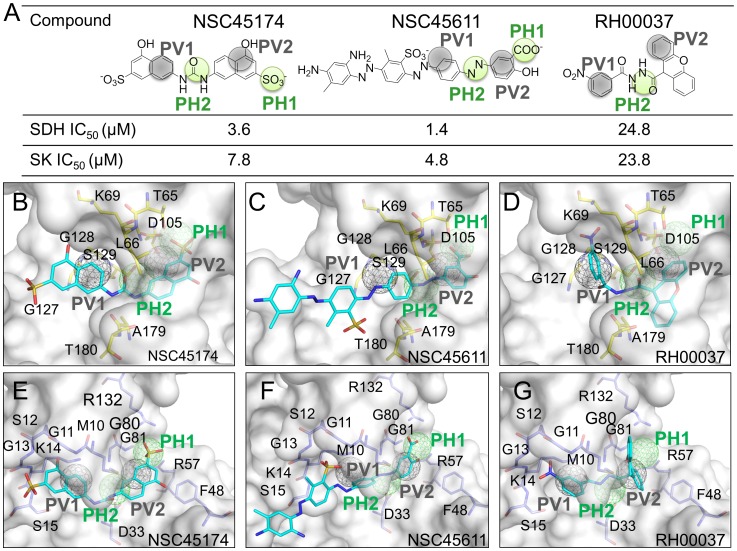
New multitarget inhibitors identified by the pathway-based screening strategy. (A) The compound structures and IC_50_ values of the multitarget inhibitors. The relationships between the inhibitors and the pathway anchors are represented by green (hydrogen-bonding interactions) and gray (van der Waals interactions) circles. Docking poses of these inhibitors and anchor residues of (B–D) SDH and (E–G) SK.

Three multitarget inhibitors that simultaneously inhibit SDH and SK were identified based on the PathSiMMap scores. Two inhibitors NSC45174 and NSC45611, match the four pathway anchors in both targets (Fig. 4) and their IC_50_ values were consistently <10 µM. The inhibitor RH00037 lacks a polar moiety near the PH1 anchor, resulting in poor IC_50_ values (24.8 µM for SDH and 23.8 µM for SK) (Fig. 4A). The sulfonate group of NSC45174 and the carboxyl group of NSC45611 form hydrogen bonds with the residues of the PH1 anchor in the same way as the hydroxyl group of shikimate in SDH and the carboxyl groups of shikimate in SK. The elimination of polar moieties in RH00037 causes an approximately 10-fold reduction in inhibitory ability, revealing the importance of the PH1 anchor for multitarget inhibitor design.

Although the urea moiety of NSC45174 is different from the azo moieties of NSC45611 and RH00037, these moieties consistently form hydrogen-bonding interactions with the pocket of the PH2 anchor (Fig. 4). NSC45174 uses naphthalene, whereas NSC45611 and RH00037 use aromatic moieties to make van der Waals contacts with the residues of the PV1 anchor. Similarly, NSC45174, NSC45611, and RH00037 use naphthalene, aromatic ring, and 9H-xanthene to make van der Waals contacts with the residues of the PV2 anchor, respectively. These ring moieties can consistently engage in van der Waals interactions with residues of PV1 and PV2 despite their differing moieties. In these inhibitors, the presence of different moieties with similar physico-chemical properties reveals the advantages of the PathSiMMaps for identifying diverse multitarget inhibitors and providing an opportunity for lead optimization.

We further carried out experiments to compare three dose-response curves (Fig. S4): (1) shikimate dehydrogenase (SDH) activity; (2) shikimate kinase (SK) activity; and (3) dual enzyme (SDH and SK) activity. The dual enzyme assay is based on the determination of the release of ADP from the substrate 3-dehydroshikimate in the presence of two enzymes. For the inhibitors (NSC45611 and NSC45174) that simultaneously blocked SDH and SK, it was interesting that the dual enzyme curve had the median effect. At inhibitor concentrations greater than the IC_90_ value, it was intriguing that the dual enzyme curves swiftly approached approximately 0, revealing the greater combined inhibitory effect. In contrast, there were nearly identical profiles for the SK-specific inhibitor (NSC162535 [14]).

The proteins share similarities in physicochemical properties and shapes of their localized binding sites, despite low sequence or low overall structural similarities. This provides an opportunity to design multitarget inhibitors or results in unexpected side effects. For complex diseases such as cancer, diabetes, and cardiovascular diseases, the inhibition of multiple proteins is necessary for efficient therapy. Current therapeutic strategies use drug combination for these diseases, which frequently results in unwanted side effects. Our studies reveal that the anchor-based alignment method can be applied to measure binding environment similarities between proteins instead of relying on sequence or structure alignments.

### Evolutionary conservation of pathway anchors

**Figure 5:**
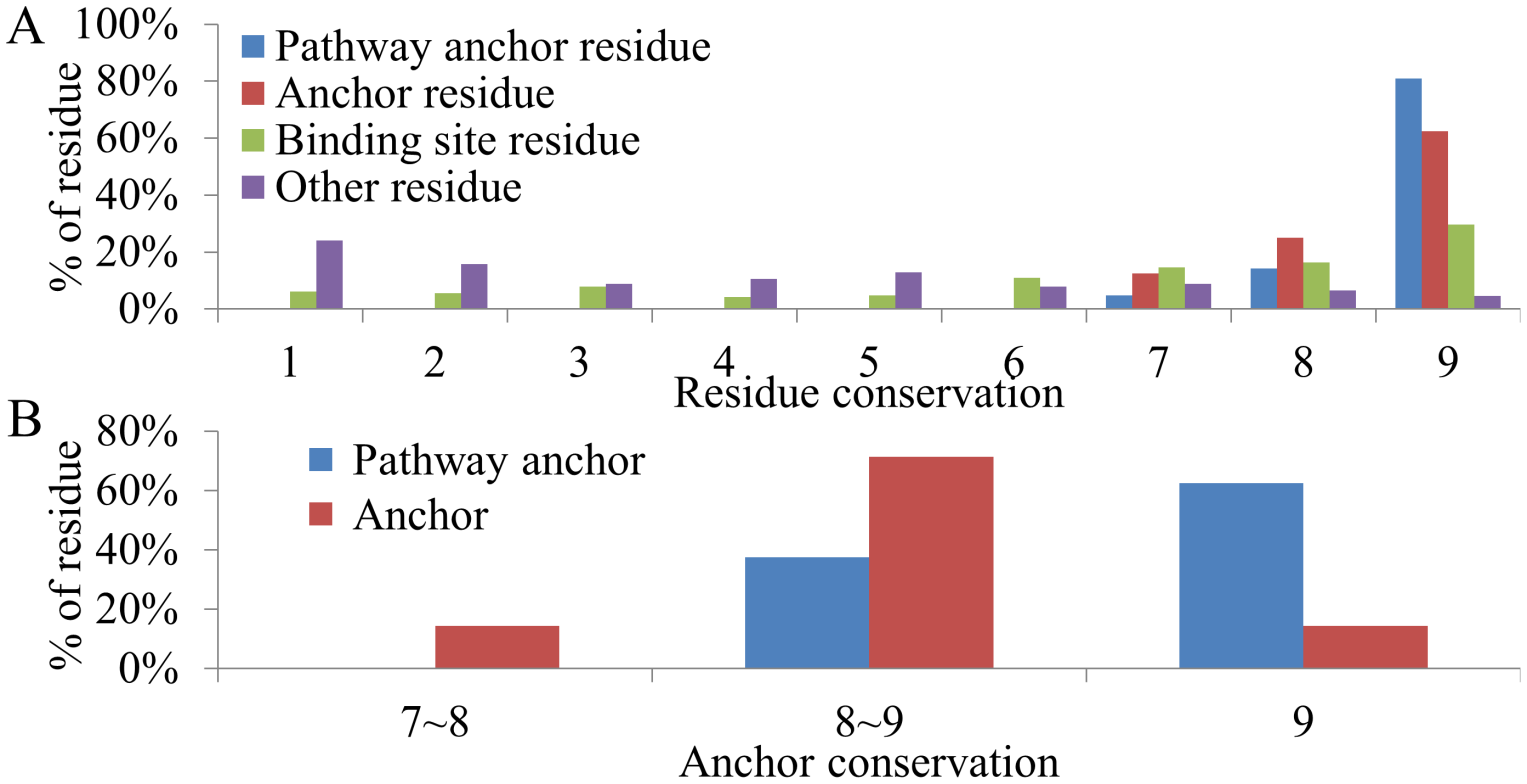
Comparison of conservation scores. (A) Conservation-score distribution of pathway anchor residues, anchor residues, binding site residues, and other residues. The scores are from 1 (least conserved) to 9 (most conserved). (B) Conservation-score distribution of pathway anchors and anchors. A conservation score of a pathway anchor or an anchor is derived by averaging the conservation scores of pathway anchor residues or anchor residues.

We further examined the pathway anchors with respect to residue conservation (Fig. 5). The residues of SDH and SK were classified into four groups: pathway anchor residues, anchor residues, binding site residues, and other residues according to the following rules. The residues of the pathway anchors were classified as pathway anchor residues. The residues that formed anchors but were not pathway anchor residues were classified as anchor residues. The residues of the defined binding sites that were neither pathway anchor nor anchor residues were classified as binding site residues. The remaining residues were classified as other residues. Each residue position was assigned an evolutionary conservation score according to the Consurf server [32]. For a query protein, the Consurf server provided a multiple sequence alignment of its homologous sequences for measuring the conservation degree of each residue position. The conservation degree was divided into nine grades. Residues with the highest conservation score, 9, represented the highly conserved positions, which often play important roles for maintaining protein functions/structure during the evolutionary process. The statistical results revealed that the pathway anchor residues are the most conserved among the four groups (Fig. 5A). The conservation score of 9 was observed for 81% of pathway anchor residues, 63% of anchor residues, 30% of binding site residues, and 5% of other residues. When we calculated an average conservation score for each anchor and pathway anchor, the pathway anchors proved to be more conserved than the anchors (Fig. 5B). For example, the conservation score for the PH1 anchor is 9, and the conservation score for each of its residues (T69, K69, and D105 in SDH; R57 and R132 in SK) is 9. The high conservation of the pathway anchors implies that they have been essential for a series of catalytic reactions during evolution owing to their importance for interacting with shikimate. This is based on structure complex observations (Fig. 3).

One of the advantages of the pathway-based screening strategy is to design multiple-target inhibitors that occupy the pathway anchors for reducing the probability of drug resistance. For multitarget inhibitors, the probability of simultaneously arising resistant mutations is exponentially lower than that of any single mutation. In contrast, the conventional strategy for developing drugs is easily susceptible to resistant mutations using a “one-disease, one-target, one-drug” strategy. The conventional strategy is ineffective against diseases with high mutation rates, such as influenza virus, cancers, and human immunodeficiency virus type 1 [7], [33], [34]. Therefore, the pathway-based screening strategy is useful for designing multitarget inhibitors for such diseases.

### A specific site and inhibitors for SDH

**Figure 6:**
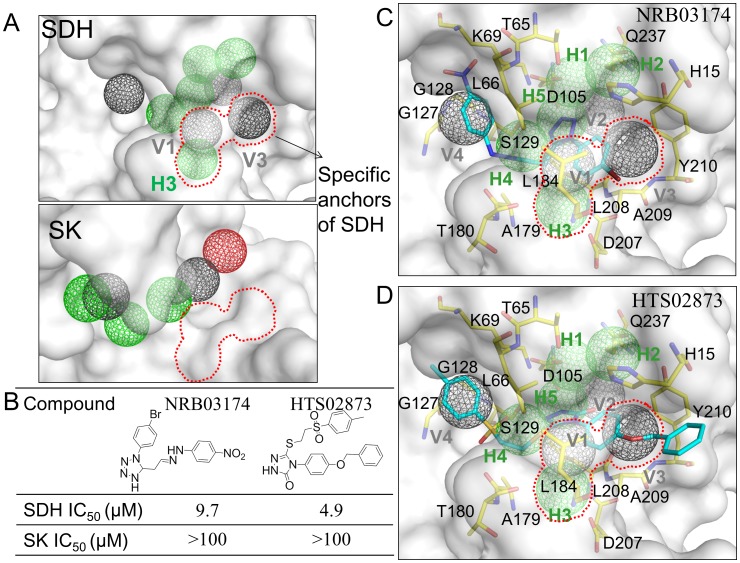
Specific anchors and selective inhibitors of shikimate dehydrogenase (SDH). (A) Specific anchors of SDH. The specific site includes these anchors H3, V1, and V3. (B) Compound structures and IC_50_ values of two selective inhibitors. Docking poses of these two selective inhibitors (C) NRB03174 and (D) HTS02873.

The alignment of the site-moiety maps of SDH and SK revealed a specific site for SDH despite many similarities shared by the two targets (Fig. 6A). The specific site consists of the H3, V1, and V3 anchors, which are not involved in the NADPH and shikimate binding sites. The specific site provided an opportunity to discover selective inhibitors for SDH. We evaluated this concept using two selective inhibitors (NRB03174 and HTS02873) that occupied three-specific anchors with high PathSiMMap scores (Fig. 6B). NRB03174 and HTS02873 inhibited SDH with IC_50_ values 9.7 µM and 4.9 µM, respectively, whereas they demonstrated no inhibitory effect at 100 µM for SK (Fig. 6B). NRB03174 interacts with the residues of the V1 and V3 anchors using the bromobenzene moiety (Fig. 6C); similarly, HTS02873 makes van der Waals contacts with the residues of the V1 and V3 anchors using the anisole moiety (Fig. 6D). Although no hydrogen-bonding interactions were observed in the specific anchors of SDH for the NRB03174/HTS02873 molecules, these two inhibitors formed hydrogen-bonding interactions with the anchor residues of the pathway anchors. For example, NRB03174 yielded hydrogen bonds with the anchor residues (L66, K69, S129, and A179), and HTS02873 made hydrogen-bonding interactions with the residues (S129, and A179).

Designing selective inhibitors for disease-specific proteins can prevent unexpected side effects that are major obstacles in clinical trials and often result in treatment failure. For example, more than 100 p38 MAP kinase inhibitors that were designed for treating inflammatory or cardiovascular diseases were suspended because of their serious side effects [35]. The above results suggested that specific anchors and the pathway anchors can be used to design selective inhibitors and multitarget inhibitors, respectively. Thus the concept of the pathway-based screening strategy can be further extended to design multitarget inhibitors of disease-specific proteins. By combining specific anchors and the pathway anchors of multiple disease-related proteins, it is possible to design multitarget inhibitors that bind disease-specific but not non-specific proteins. Such multitarget inhibitors can enhance therapeutic potency and minimize side effects.

### Performance and profile analyses

**Figure 7:**
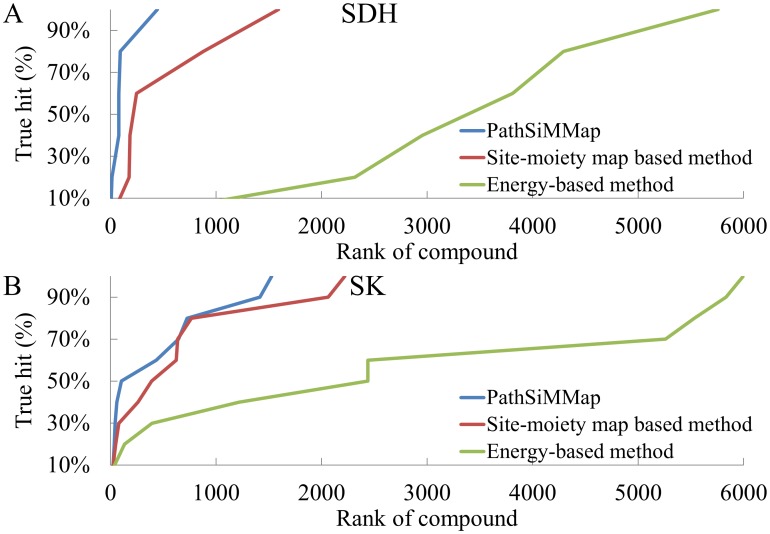
Performance of the PathSiMMap compared with site-moiety map-based and energy-based methods for (A) SDH and (B) SK. The PathSiMMap (blue line) is the best and significantly outperforms the site-moiety map (red line) and the energy-based method (green line) for identifying SDH and SK inhibitors.

The accuracy of the PathSiMMap was assessed using the hit rate and compared with site-moiety map and energy-based methods. The energy-based method used here was the piecewise linear potential (PLP) of GEMDOCK [20]. GEMDOCK is comparable to some docking methods (e.g., DOCK, FlexX, and GOLD) on the 100 protein-ligand complexes and has similar accuracy to some energy-based scoring functions in the prediction of binding affinities [20], [24]. During the docking process, GEMDOCK first assigned formal charge and atom type (*i.e.*, donor, acceptor, both, or nonpolar) to atoms of compounds and proteins. Then, the GEMDOCK PLP measures intermolecular potential energy between proteins and docked compounds. The intermolecular potential energy includes electrostatic, van der Waals, and hydrogen-bonding interactions. The compounds can be ranked based on their intermolecular potential energy. The hit rate is defined as *A_h_*/*T_h_* (%), where *A_h_* is the number of active compounds among the *T_h_* highest-ranking compounds. For SDH, the active compounds used for verification were the three multitarget inhibitors and the two specific inhibitors (*A_h_* = 5). For SK, the active compounds used for verification were the seven SK inhibitors [14] (Fig. S5), and three multitarget inhibitors (*A_h_* = 10). The hit rate of the PathSiMMap was considerably better than that of other methods used for identifying inhibitors of SDH and SK (Fig. 7, Tables S1 and S2). Our pathway-based screening strategy can be used to enhance the hit rate because the pathway anchors are often highly conserved and important for biological functions (Figs. 3 and 5). This suggests that the pathway anchors often play important roles for ligand binding. Thus, the compounds that match the pathway anchors are often potential inhibitors of the target proteins. For example, for SDH, the ranks of NSC45174 were 3810 by the energy-based method, 177 by the site-moiety map, and 13 by PathSiMMap.

We selected 20 compounds (Tables S3 and S4) for bioassay based on their PathSiMMap scores, drug-like properties, availabilities, and domain knowledge. We performed the compound-anchor profile analysis to find why NSC45174 and NSC45611 were more potency than other top-ranked compounds (Fig. S6). This profile analysis showed that NSC45174 and NSC45611 simultaneously matched the four pathway anchors of SDH and SK (Fig. S6A) and inhibited them with IC_50_ values ≦10 µM. In contrast, most of the inactive compounds matched 2–3 pathway anchors of SDH and SK. For example, KM02359 has no polar moieties to yield hydrogen-bonding interactions with the PH1 anchor residues of SDH and SK (Figs. S6A and S6C). CD01870 lacks a polar moiety in the PH1 anchor and is unable to form hydrogen bonds with the anchor residues of SDH and SK (Figs. S6A and S6D).

We next analyzed the compound–residue interaction profiles to find why some compounds that matched the four pathway anchors were inactive for both SDH and SK (Fig. S6B). These profiles showed that NSC45174, NSC45611, and RH00037 maintained the conserved interactions (i.e., those commonly found with >50% of inhibitors) with the anchor residues of SDH and SK (e.g., K69, D105, G127, A179, L208, and S129 in SDH; M10, G11, S12, G13, K14, S15, D33, R57, G80, and R132 in SK). These conserved interactions of the pathway anchors may have accounted for the potency of NSC45174 and NSC45611. These profiles indicated that some compounds (e.g. HTS05470) with high PathSiMMap scores lacked several of the conserved interactions, which may have resulted in their inactivity. For example, HTS05470 lost the conserved hydrogen-bonding interactions with these residues (A179 and L208 in SDH; K14 and S15 in SK) (Figs. S6B and S6E). According to both compound-anchor profiles and compound–residue interaction profiles, these results showed that the compound often inhibits proteins when it highly matches the pathway anchors and keeps conserved interactions. In addition, we applied the pathway-based screening strategy for additional four pathways (Figs. S7, S8, S9, S10, and Text S1).

### Pathway site-moiety map of seven proteins in shikimate pathway

The pathway-based screening strategy to discover multitarget inhibitors relies on the following criteria: (1) the proteins catalyze ligands with common substructures, and (2) these proteins share conserved binding environments and comparable anchors in their site-moiety maps. We selected the other five proteins in the shikimate pathway of *Helicobacter pylori* to examine whether they share conserved binding environments (i.e. pathway anchors) with SDH and SK (Fig. S11). These proteins include DAHP synthase, 3-dehydroquinate synthase (3CLH), 3-dehydroquinate dehydratase (1J2Y), EPSP synthase, and chorismate synthase (1UM0). Because structures of DAHP synthase and EPSP synthase are unavailable, we obtained their structures using an in-house homology-modeling server [36]. First, the site-moiety maps of these five proteins were established. The anchor-based alignment method was then applied to identify the pathway anchors of these seven proteins. Among these proteins, 3-dehydroquinate synthase, SDH, SK, and EPSP synthase share the four pathway anchors (Fig. S11). The former three proteins have similar substrates (DAHP, 3-dehydro shikimate, and shikimate) and cofactors (NAD^+^, NADPH, and ATP) (Fig. S1). Conversely, the PEP, the cofactor of EPSP synthase, is much smaller than NAD^+^, NADPH, or ATP.

These four pathway anchors located across substrate and cofactor sites often play key roles in catalytic reactions and ligand bindings for 3-dehydroquinate synthase, SDH, SK, and EPSP synthase (Figs. 3 and S12). 3-dehydroquinate synthase converts DAHP into DHQ with the cofactor NAD^+^ (Fig. S1). The PH1 anchor of 3-dehydroquinate synthase is situated at the DAHP site (Fig. S12), while the PH2, PV1, and PV2 sit at the NAD^+^ site. Three polar residues (D126, K210, and R224) comprise the PH1 anchor. The carboxyl moiety of DAHP forms hydrogen-bonding interactions with the PH1 anchor residues (K210 and R224), involving in the catalytic reaction [37]. The nicotinamide moiety of NAD^+^ interacts with the PH2 anchor residue (D99) and the PV2 anchor residues (D126, K132, and K210) by hydrogen-bonding and van der Waals interactions, respectively. Two residues (G95 and L122) constitute the PV1 anchor and make van der Waals interactions with the tetrahydrofuran-3,4-diol moiety of NAD^+^. EPSP synthase catalyzes the conversion of shikimate-3-phosphate into EPSP with PEP (Fig. S1). The PH1 anchor of EPSP synthase consists of three residues (A154, S155, and K329). A hydrogen bonding network is formed between the anchor residues (S155 and K329) and the phosphate moiety of shikimate-3-phosphate. Three polar residues comprise (K11, T83, and D302) the PH2 anchor, and these residues yield hydrogen bonds with the phosphate moiety of PEP and the hydroxyl moiety of shikimate-3-phosphate. The PV1 anchor consists of three residues with long side chains, including K11, D302, and E330. The acrylic acid moiety of PEP is situated at this anchor, and makes van der Waals interactions with these residues. The cyclohexene moiety of shikimate-3-phosphate is sandwiched between the PV2 anchor residues (Q157, R182, and I301) and forms stacking interactions with them. These observations show the importance of these pathway anchors for performing biological functions of these proteins. In addition, although these four proteins have different functions, their pathway anchor residues have similar physicochemical properties for interacting their substrates and cofactors. For example, the PH1 anchor residues of 3-dehydroquinate synthase, SDH, SK, and EPSP synthase are polar and consistently form hydrogen bonding interactions with carboxyl, ketone, carboxyl, and phosphate moieties of their substrates, respectively.

We then docked the multitarget inhibitors of SDH and SK into 3-dehydroquinate synthase and EPSP synthase to examine whether these inhibitors match the pathway anchors of these two proteins. The docked poses show that NSC45174 matches the four pathway anchors in 3-dehydroquinate synthase, while NSC45611 and RH00037 match three pathway anchors (Fig. S13). The docked pose of NSC45174 in 3-dehydroquinate synthase is similar to those in SDH and SK. For example, the sulfonate moiety of NSC45174 is located at the PH1 anchor of these three proteins and consistently forms hydrogen bonds with the PH1 anchor residues (Figs. 4B, 4E, and S13A). Similarly, the naphthalene moiety of NSC45174 consistently sits at the PV2 anchor, and makes van der Waals interactions with the anchor residues. In contrast, these three compound match 2–3 pathway anchors in EPSP synthase. For instance, the sulfonate moiety of NSC45174 is located at the PV1 anchor and thereby is unable to form hydrogen-bonding interactions with the PH2 anchor residues (Fig. S13D). Next, we carried out experiments to determine IC_50_ values of the three compounds for 3-dehydroquinate synthase. NSC45174 inhibited 3-dehydroquinate synthase with an IC_50_ value 7.1 µM, while NSC45611 and RH00037 showed no inhibitions (Figs. S13G and S13I). NSC45174 is a novel multitarget inhibitor that simultaneously inhibited three proteins (SDH, SK, and 3-dehydroquinate synthase) of the shikimate pathway. These results reveal that the pathway-based screening strategy can identify multitarget inhibitors in a pathway.

## Materials and Methods

### Preparations of protein structures and screening databases

Apo-form structures of SDH and SK were selected for virtual screening because the use of closed-form structures induced by bound ligands may limit the diversity of identified inhibitors. For defining binding sites, the apo-form structures of SDH (3PHG) and SK (1ZUH [19]) were aligned to their respective closed-form structures SDH (3PHI) and SK (1ZUI [19]), using a structural alignment tool [38]. The bound ligands (shikimate and NADPH for SDH and shikimate and phosphate for SK) were used to determine the binding sites of SDH and SK. The binding sites of these structures were defined by residues situated ≤8 Å from the bound ligands.

We selected compounds from two public databases, Maybridge and National Cancer Institute, to generate the PathSiMMaps and discover multitarget inhibitors because of their rapid availability and low cost. Compounds with molecular weight <200 or >650 daltons were not selected. The total number of compounds selected for screening was 302,909.

### Computational screening and establishment of site-moiety maps

The 302,909 compounds were docked into the binding sites of SDH and SK using an in-house docking tool, GEMDOCK [20] (Fig. S14A) to establish the site-moiety maps of target proteins. Subsequently, the top 2% compounds (approximately 6,000) ranked by docking energy were selected to establish site-moiety maps. We inferred site-moiety maps to recognize interaction preferences between binding pockets and moieties using the top-ranked 2% compounds. First, protein-compound interaction profiles were generated based on the PLP calculated by GEMDOCK (Fig. S14B). The profiles described the interactions (*i.e.*, E, H, and V interactions) between the compounds and the protein residues. Each profile can be represented by a matrix with size *P*×*C*, where *P* and *C* are the numbers of docked compounds and interacting residues of a protein. For the E and H profiles, the entry was set to 1 (green regions in Fig. S14B) if the compound forms electrostatic or hydrogen-bonding interactions with the residues such as T65, K69, and D105 in the anchor H1; otherwise, the entry was set to 0 (black regions). For the V profile, the entry was set to 1 if the V energy was less than −4 kcal/mol.

The consensus interacting residues (*e.g.*, T65, K69, and D105) of the profiles recognized according to Z scores often play key roles in biological functions. For each profile, the Z score (*Z_i_*) of the protein residue *i* was computed by 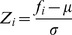, where *f_i_* is the observed interaction frequency between compounds and residue *i*, and *μ* and *σ* are the mean and the standard deviation of interaction frequency derived from 1,000 randomly shuffled profiles. We considered the residue *i* to be a consensus interacting residue if its Z score was greater than 1.645, a common threshold used in statistics (corresponding to a 95% confidence level). Then spatially neighboring interacting residues and their interactive moieties with statistical significance were assigned as an anchor (Fig. S14C). Finally, the site-moiety map of each target was constructed (Fig. S14D).

### Establishment of pathway site-moiety maps and identification of pathway inhibitors

Pathway anchors, which are conserved anchors among the target proteins, represent key features including consensus interactions between the compounds and the binding pockets in a pathway (Figs. 2 and 3). Identifying pathway anchors using a structural alignment tool is a challenging task because of low sequence identity (8.3%) and structure similarity (RMSD is 4.8 Å) between SDH and SK [38]. To address this task, we developed an anchor-based alignment method according to spatial arrangements, the interaction-type similarity, and the volume similarity of the aligned anchors (Fig. S15). Each aligned anchor pair *x* between SDH and SK site-moiety maps is assigned an anchor alignment score (*AAS*(*x*)), which is defined as
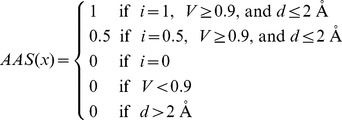
where *i* is interaction-type similarity, *V* is anchor-volume similarity, and *d* is the distance between the aligned anchors. *i* is set to 1 if the aligned anchors have the same interaction type or to 0.5 when an E anchor is aligned to an H anchor because negatively/positively charged moieties of the E anchor are able to form hydrogen bonds as well as polar moieties of the H anchor; otherwise *i* is set to 0. *V* is defined as 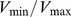, where *V*_max_ and *V*_min_ are the respective volumes of the larger and the smaller anchor. Then the alignment was achieved by maximizing the similarity score (*S*) between the site-moiety maps of SDH and SK. The similarity score is defined as 
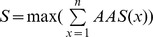
, where *n* is the number of the aligned anchors. The alignment of the two site-moiety maps was achieved by seeking the highest similarity score using exhaustively superimposing the anchors. The aligned anchors were considered to be the pathway anchors, and the center of the pathway anchor was defined as the geometric center of the two aligned anchors. These pathway anchors consisted of the PathSiMMaps of SDH and SK (Fig. S15C).

Compounds matching the pathway anchors were considered potential inhibitors for the shikimate pathway. For compound *j* at a binding site, the PathSiMMap score (*PS*), a measure of the inhibition capability, was calculated as
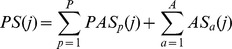
where *PAS_p_*(*j*) is the pathway anchor score of compound *j* in the pathway anchor *p*; *AS_a_*(*j*) is the anchor score of compound *j* in anchor *a*; *P* and *A* are the numbers of the pathway anchors and anchors, respectively. Here *PAS_p_*(*j*) is set to 1 if the compound *j* matches the pathway anchor *p* and otherwise to 0. Similarly, *AS_a_*(*j*) is set to 1 if the compound *j* matches the anchor *a*. For example, *P* is 4, and *A* is 9 and 6 for SDH and SK, respectively. The screening compounds were ranked based on their PathSiMMap scores for SDH and SK. Then, the compounds were re-ranked by consensus rankings of SDH and SK PathSiMMap rankings for selecting potential multitarget inhibitors. Finally, the top-ranked compounds that were commercially available were selected for bioassay. In addition, for SDH, the top-ranked compounds derived from the specific anchor were selected for bioassay. These compounds were considered to be selective inhibitors for SDH.

### Single enzyme inhibition assay and dual inhibition

The SK activity was measured by coupling the release of ADP from the SK-catalyzed reaction to the oxidation of NADH using pyruvate kinase (EC 2.7.1.40) and lactate dehydrogenase (EC 1.1.1.27) as coupling enzymes [31]. SDH activity was determined by monitoring the formation of NADPH. The initial rate of the reaction was measured by the increase in absorbance at *A*340 (ε = 6,200 M^−1^ cm^−1^) in the present of shikimate. The assay was performed at 25°C in a mixture of 100 mM Tris-HCl buffer, pH 8.0.

Both SK and SDH were used in a final enzyme concentration of 100 nM. For determination of IC_50_ for each inhibitor, the assay was initiated by the addition of shikimate (1.6 mM) after incubation in a buffer containing cofactor (2 mM ATP for SK or 2 mM NADP for SDH), enzyme, and inhibitor (dissolved in 5% dimethyl sulfoxide). All assays were conducted in a 96-well microplate and analyzed with a spectrophotometer (FLUOstar OPTIMA, BMG LABTECH). A dose-response curve was fitted using the non-linear regression function of GraphPad Prism®.

We performed ADP assay providing a direct method to analyze SDH-SK dual enzyme activity. The assay was initiated by addition of the 3-dehydroshikimate (2 mM) after incubating in a reaction mixture containing 2.5 mM ATP, 0.5 mM NADPH, 100 nM SDH, 100 nM SK enzyme, 50 mM KCl, 5 mM MgCl_2_ and different inhibitors. The reaction was carried out at 25°C in a mixture of 100 mM Tris-HCl buffer, pH 7.5 and terminated at 100°C for 5 mins in the reaction time of initial rate. The amount of ADP was measured by using ADP Colorimetric Assay Kit II (BioVision) according to the manufacturer's instruction. We also performed 3-dehydroquinate synthase inhibition assay. The reaction was comprised of 3-deoxy-D-arabinoheptulosonate 7-phosphate (1 mM) and NAD+ (0.5 mM). The amount of NADH was measured by using NAD+/NADH Quantification Kit (BioVision). All assays were conducted in a 96-well microplate and analyzed with a spectrophotometer (FLUOstar OPTIMA, BMG LABTECH). The dose-response curve was fitted using the non-linear regression function of GraphPad Prism. The IC_90_ values were computed from the IC_50_ and Hill slop.

## Supporting Information

Figure S1 **Substrate/products, cofactors, and their similarities in shikimate pathway.** (A) Proteins, metabolites, and their chemical reactions involved in the shikimate pathway. Among the metabolites, DHQ, 3-dehydroshikimate, shikimate, shikimate-3-phosphate, EPSP, and chorismate share similar scaffolds (blue parts). (B) Compound similarity matrixes of (B) substrate/products and (C) cofactors. The Similarity between two compounds is represented by a MACCS-Tanimoto value obtained from OpenBabel (http://openbabel.org/wiki/Main_Page). The substrate/products with the similar scaffolds have high MACCS-Tanimoto values (blue block). The cofactors are similar to each other with high MACCS-Tanimoto values.
(TIF)

Figure S2 **Site-moiety map of shikimate dehydrogenase.** (A) Anchors with conserved interacting residues. Hydrogen-bonding and van der Waals anchors are colored in green and gray, respectively. (B) The SDH ligands on the site-moiety map. The ligands are shikimate (one of the substrates) and NADPH (cofactor) (3PHI). (C) Moiety preferences of anchors.
(TIF)

Figure S3 **Site-moiety map of shikimate kinase.** (A) Anchors with conserved interacting residues. Negatively charged, hydrogen-bonding, and van der Waals anchors are colored in red, green, and gray, respectively. (B) Ligands of shikimate kinase on the site-moiety map. The ligands are shikimate (one of substrates) and ACP (ATP analog) (PDB code 1ZYU, a shikimate kinase structure of *Mycobacterium tuberculosis*). (C) Moiety preferences of anchors.
(TIF)

Figure S4 **Comparison of three dose-response curves of shikimate dehydrogenase (SDH), shikimate kinase (SK), and dual enzyme (SDH and SK) activities on three compounds, NSC45611, NSC45174, and NSC162535 (specific for SK).** For NSC45611 and NSC45174, at inhibitor concentrations greater than the IC_90_ value, the dual enzyme curves swiftly approached approximately 0, revealing the greater combined inhibitory effect. In contrast, there were nearly identical profiles for the SK-specific inhibitor (NSC162535).
(TIF)

Figure S5 **Inhibitors of shikimate kinase used for assessing performance.**
(TIF)

Figure S6 **Interaction profile analyses of tested compounds.** (A) Compound-anchor interaction profile of top-ranked compounds. (B) Compound-residue interaction profile of compounds matching the four pathway anchors. Docked poses of the top-ranked compounds (C) KM02359, (D) CD01870, and (E) HTS05470.
(TIF)

Figure S7 **Pathway site-moiety maps of FPPS and GGPPS in isoprenoid biosynthesis pathway.** (A) The proteins in the isoprenoid biosynthesis pathway. Among these protein, FPPS and GGPPS are directly connected. (B) Chemical reactions of FPPS and GGPPS. FPPS converts GPP to FPP using IPP, and then GGPPS converts FPP to GGPP along with IPP. The compounds (GPP, FPP, and GGPP) share the same substructure (blue region). (C) Establishment and alignment of site-moiety maps of FPPS and GGPPS. Electrostatic, hydrogen-bonding, and van der Waals anchors are colored in red, green, and gray, respectively. (D) Pathway anchors of FPPS and GGPPS. The multitarget inhibitor, minodronic acid, matched the PH1, PH2, PH3, and PV1 anchors. The docking poses in FPPS and GGPPS are represented by yellow and cyan, respectively. (E) Performance of the pathway-based screening strategy for the multitarget inhibitor compared with the site-moiety map-based and energy-based (GEMDOCK) methods.
(TIF)

Figure S8 **Pathway site-moiety maps of CAIV and CAII in proximal tubule bicarbonate reclamation pathway.** (A) The proteins in the proximal tubule bicarbonate reclamation pathway. CAIV and CAII are directly connected. (B) Chemical reactions of CAIV and CAII. CAIV catalyzes HCO_3_^−^ and H^+^ into H_2_O and CO_2_. CAII then converts CO_2_ into HCO_3_^−^. (C) Establishment and alignment of site-moiety maps of CAIV and CAII. Hydrogen-bonding and van der Waals anchors are colored in green and gray, respectively. (D) Pathway anchors of CAIV and CAII The multitarget inhibitor, NCX265, matched the PE1, PH1, PV1, and PV2 anchors. The docking poses of NCX265 in CAIV and CAII are represented by pink and purple, respectively. (E) Performance of the pathway-based screening strategy for the multitarget inhibitor compared with the site-moiety map-based and energy-based (GEMDOCK) methods.
(TIF)

Figure S9 **Pathway site-moiety maps of DHFR and TS in one carbon pool by folate pathway.** (A) The proteins in the one carbon pool by folate pathway. Among these proteins, DHFR and TS are connected in the pathway. (B) Chemical reactions of DHFR and TS. DHFR catalyzes DHF to THF by NADPH. TS converts 5,10-CH2-THF to DHF using dUMP. These compounds share similar scaffolds. (C) Establishment and alignment of the site-moiety maps of DHFR and TS. Electrostatic, hydrogen-bonding, and van der Waals anchors are colored in red, green, and gray, respectively. (D) Pathway anchors of DHFR and TS. Methotrexate matched the PE1, PV1, PV2, and PV3 anchors. The docking poses of Methotrexate in DHFR and TS are represented by pink and yellow, respectively. (E) Performance of the pathway-based screening strategy for the multitarget inhibitor compared with the site-moiety map-based and energy-based (GEMDOCK) methods.
(TIF)

Figure S10 **Pathway site-moiety maps of ALDH and AKR in retinoid metabolic pathway.** (A) The proteins in the retinoid metabolic pathway. Among these proteins, ALDH and AKR are indirectly connected in the branched pathway. (B) Chemical reactions of ALDH and AKR. The former converts retinaldehyde into retinoic acid by NAD^+^. The latter catalyzes retinaldehyde into retinol using NADPH. (C) Establishment and alignment of the site-moiety maps of ALDH and AKR. Electrostatic, hydrogen-bonding, and van der Waals anchors are colored in red, green, and gray, respectively. (D) Pathway anchors of ALDH and AKR. Their common inhibitor, 7-hydroxy-4-phenylcoumarin, matched the PH1, PV1, and PV2 anchors. The docking poses of 7-hydroxy-4-phenylcoumarin in ALDH and AKR are represented by yellow and cyan, respectively. (E) Performance of the pathway-based screening strategy for the multitarget inhibitor compared with the site-moiety map-based and energy-based (GEMDOCK) methods.
(TIF)

Figure S11 **Anchor alignment for pathway anchors of seven proteins in shikimate pathway.** (A) Site-moiety maps of DAHP synthase (template 2B70), 3-dehydroquinate synthase (3CLH), 3-dehydroquinate dehydratase (1J2Y), shikimate dehydrogenase (3PHG), shikimate kinase (1ZUH), EPSP synthase (template 1RF6), and chorismate synthase (1UM0). Negatively charged, hydrogen-bonding, and van der Waals anchors are colored in red, green, and gray, respectively. (B) Aligned anchors of the seven site-moiety maps (sphere) and the pathway anchor (mesh). (C) Anchor profile of the seven proteins. A cell is colored in green if the protein has the pathway anchor; otherwise the region is colored in black.
(TIF)

Figure S12 **Pathway site-moiety map of 3-dehydroquinate synthase and EPSP synthase.** Relationship between the pathway anchors and substrate/cofactor for (A) 3-dehydroquinate synthase and (B) EPSP synthase. The ligands of 3-dehydroquinate synthase are CRB (DAHP analog) (PDB code 1DQS, a 3-dehydroquinate synthase structure of *Emericella nidulans*) and NAD^+^. The EPSP synthase of *Helicobacter pylori* was modeled using a template structure (PDB code 1RF6). The ligands of EPSP synthase are shikimate-3-phosphate and PEP (PDB code 2O0E, an EPSP synthase structure of *Mycobacterium tuberculosis*). Hydrogen-bonding interactions between ligand and pathway anchor residues are represented as green dashes. (C) Pathway anchor residues of 3-dehydroquinate synthase and EPSP synthase.
(TIF)

Figure S13 **Relationship between pathway anchors and docking poses of NSC45174, NSC45611, and RH00037 for 3-dehydroquinate synthase and EPSP synthase.** Docking poses of these compounds and anchor residues of (A–C) 3-dehydroquinate synthase and (D–F) EPSP synthase. Compound-pathway anchor profiles of (G) 3-dehydroquinate synthase and (H) EPSP synthase. A cell is colored in green if the compound matches the pathway anchor; otherwise the region is colored in black. (I) Dose-response curve of 3-dehydroquinate synthase activity on NSC45174.
(TIF)

Figure S14 **Establishment of a site-moiety map for a protein binding site using shikimate dehydrogenase.** (A) Molecular docking for the screening target. (B) Merged protein–compound interaction profiles including electrostatic, hydrogen-bonding, and van der Waals profiles. A cell is colored in green if there is interaction (electrostatic, hydrogen-bonding, or van der Waals) between a compound moiety and a residue; otherwise the region is colored in black. (C) An anchor of the site-moiety map shown as an example. An anchor includes conserved interacting residues, moiety preferences, and interaction type. The example is hydrogen-bonding anchor, including a binding pocket consisting of polar residues, T65, K69, and D105. (D) Site-moiety map of SDH. The map consists of five hydrogen-bonding anchors (H1–H5), and four van der Waals anchors (V1–V4). Hydrogen-bonding and van der Waals anchors are colored in green and gray, respectively.
(TIF)

Figure S15 **Identification of pathway anchors using the anchor-based alignment method.** (A) Site-moiety maps of SDH and SK. (B) Alignment process and aligned anchors. SDH anchors V4, H4, H5, and H1 were aligned to SK anchors V1, H3, V2, and E1, respectively. (C) Pathway anchors of SDH and SK.
(TIF)

Table S1 **Tested compound ranks of the SDH inhibitors.**
(DOC)

Table S2 **Tested compound ranks of the SK inhibitors.**
(DOC)

Table S3 **PathSiMMap ranks and IC_50_ values of the tested multitarget compound candidates.**
(DOC)

Table S4 **PathSiMMap ranks and IC_50_ values of the tested SDH-specific compound candidates.**
(DOC)

Table S5 **Selected pathways, proteins, and multitarget inhibitors used for verifying the pathway-based screening strategy.**
(DOC)

Text S1 **Application of pathway-based screening strategy on four pathways.**
(DOC)
