# Supplementary figures and images for: Pathway-based Screening Strategy for Multitarget Inhibitors of Diverse Proteins in Metabolic Pathways
Source: PLoS Comput Biol. 2013 Jul 4;9(7):e1003127. doi: 10.1371/journal.pcbi.1003127 (PMC3701698; doi:10.1371/journal.pcbi.1003127)

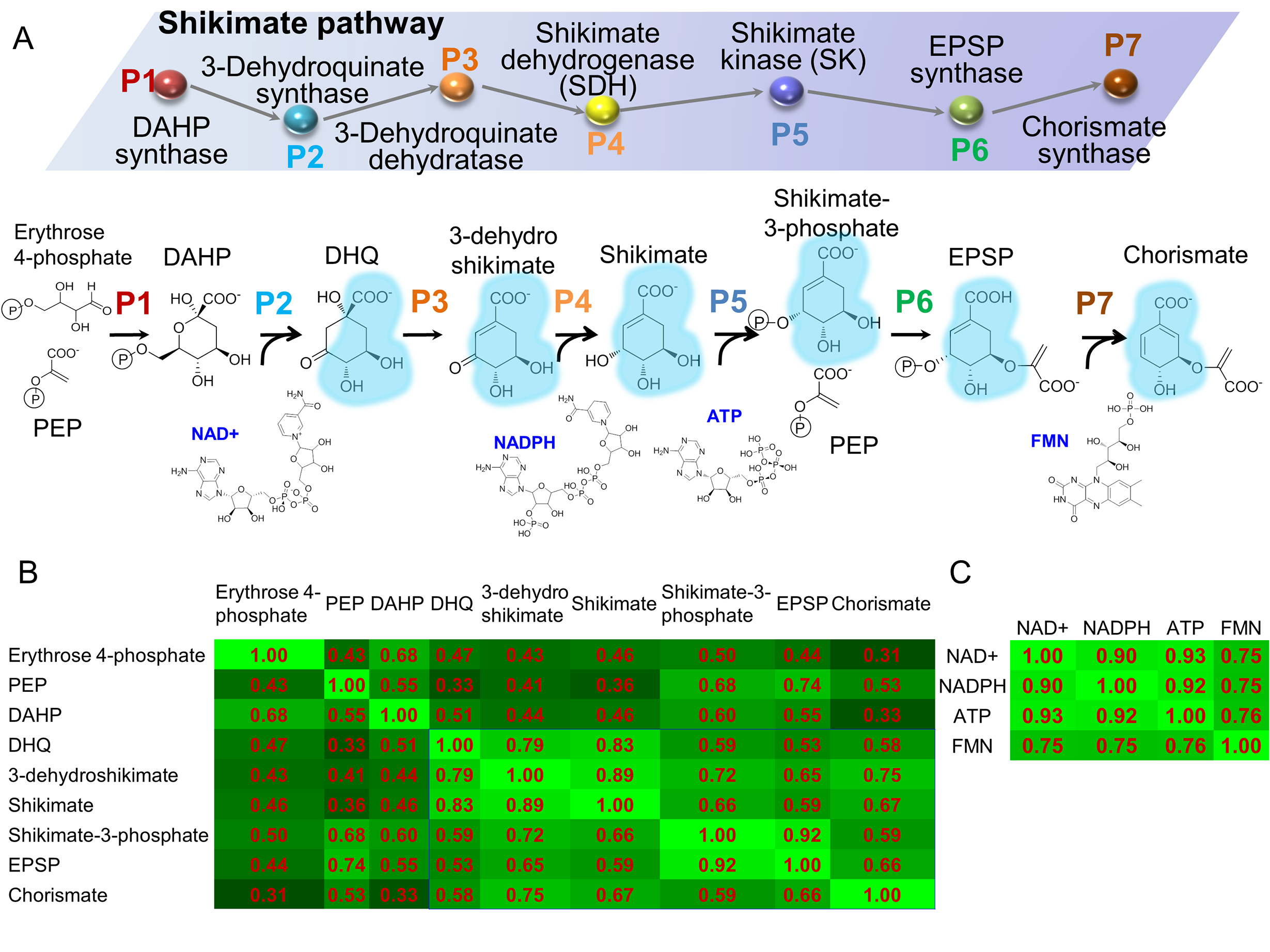

Supplement: Figure S1 — Substrate/products, cofactors, and their similarities in shikimate pathway. (A) Proteins, metabolites, and their chemical reactions involved in the shikimate pathway. Among the metabolites, DHQ, 3-dehydroshikimate, shikimate, shikimate-3-phosphate, EPSP, and chorismate share similar scaffolds (blue parts). (B) Compound similarity matrixes of (B) substrate/products and (C) cofactors. The Similarity between two compounds is represented by a MACCS-Tanimoto value obtained from OpenBabel (http://openbabel.org/wiki/Main_Page). The substrate/products with the similar scaffolds have high MACCS-Tanimoto values (blue block). The cofactors are similar to each other with high MACCS-Tanimoto values. (TIF) [file pcbi.1003127.s001.tif]

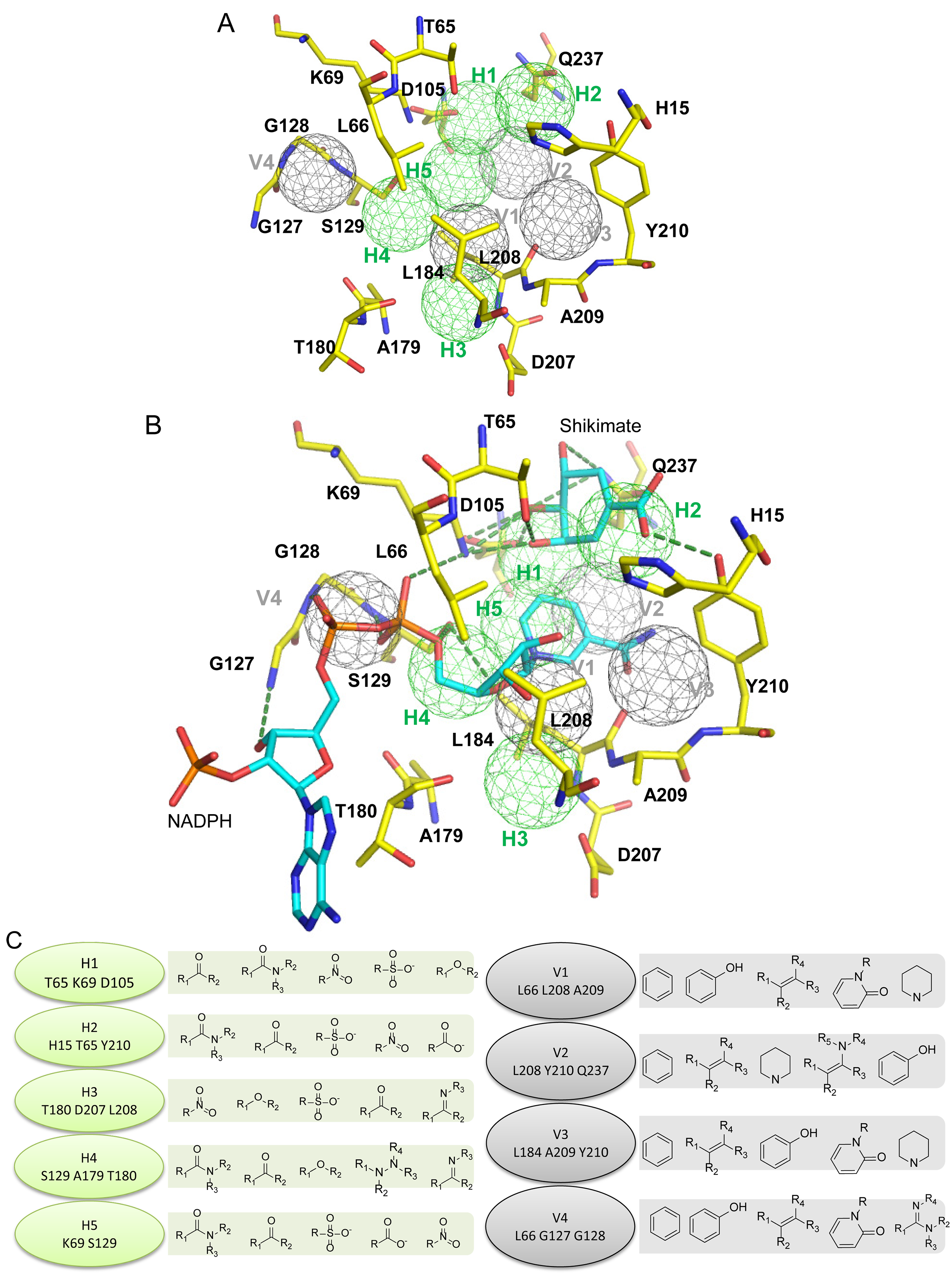

Supplement: Figure S2 — Site-moiety map of shikimate dehydrogenase. (A) Anchors with conserved interacting residues. Hydrogen-bonding and van der Waals anchors are colored in green and gray, respectively. (B) The SDH ligands on the site-moiety map. The ligands are shikimate (one of the substrates) and NADPH (cofactor) (3PHI). (C) Moiety preferences of anchors. (TIF) [file pcbi.1003127.s002.tif]

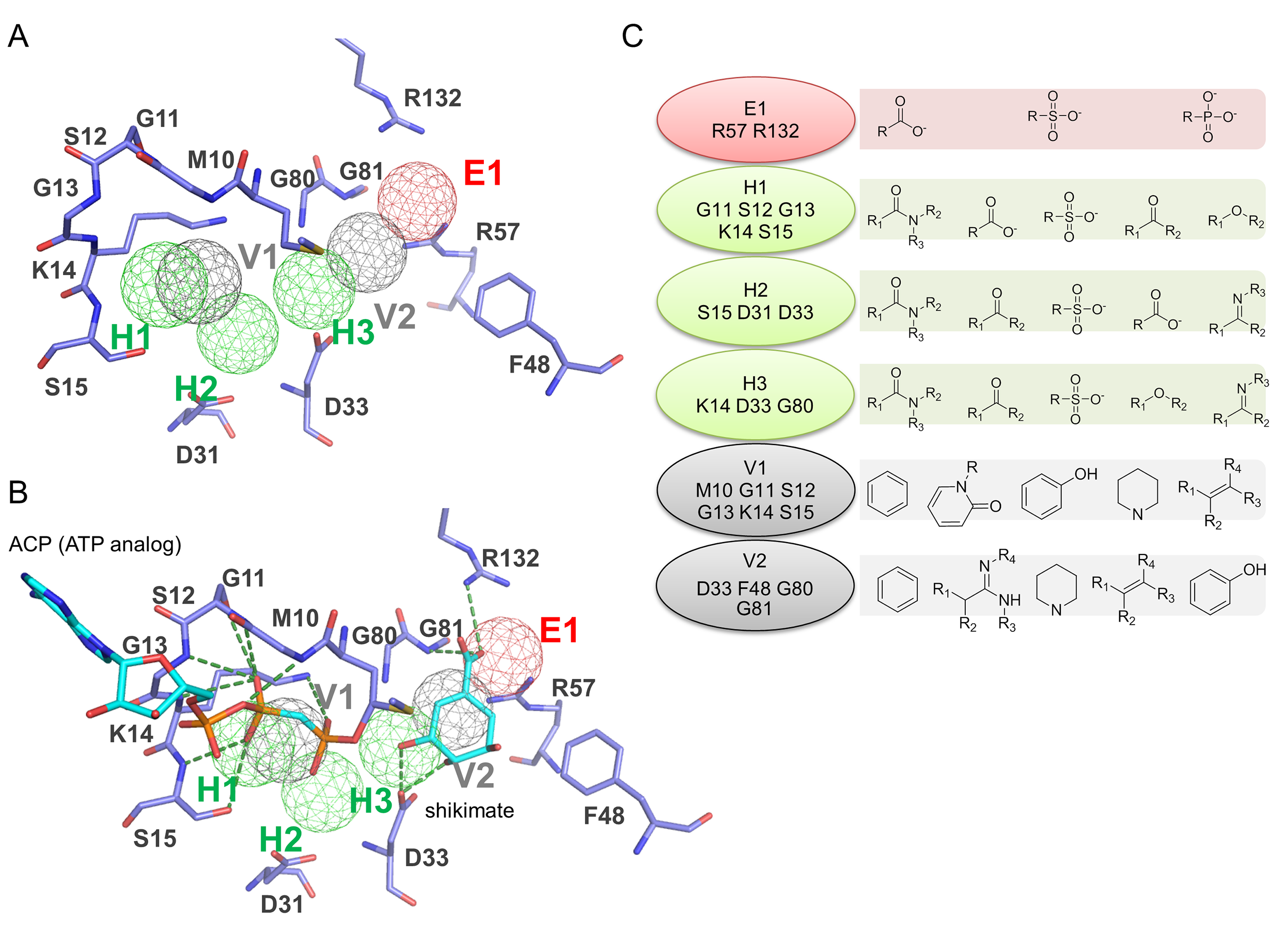

Supplement: Figure S3 — Site-moiety map of shikimate kinase. (A) Anchors with conserved interacting residues. Negatively charged, hydrogen-bonding, and van der Waals anchors are colored in red, green, and gray, respectively. (B) Ligands of shikimate kinase on the site-moiety map. The ligands are shikimate (one of substrates) and ACP (ATP analog) (PDB code 1ZYU, a shikimate kinase structure of Mycobacterium tuberculosis). (C) Moiety preferences of anchors. (TIF) [file pcbi.1003127.s003.tif]

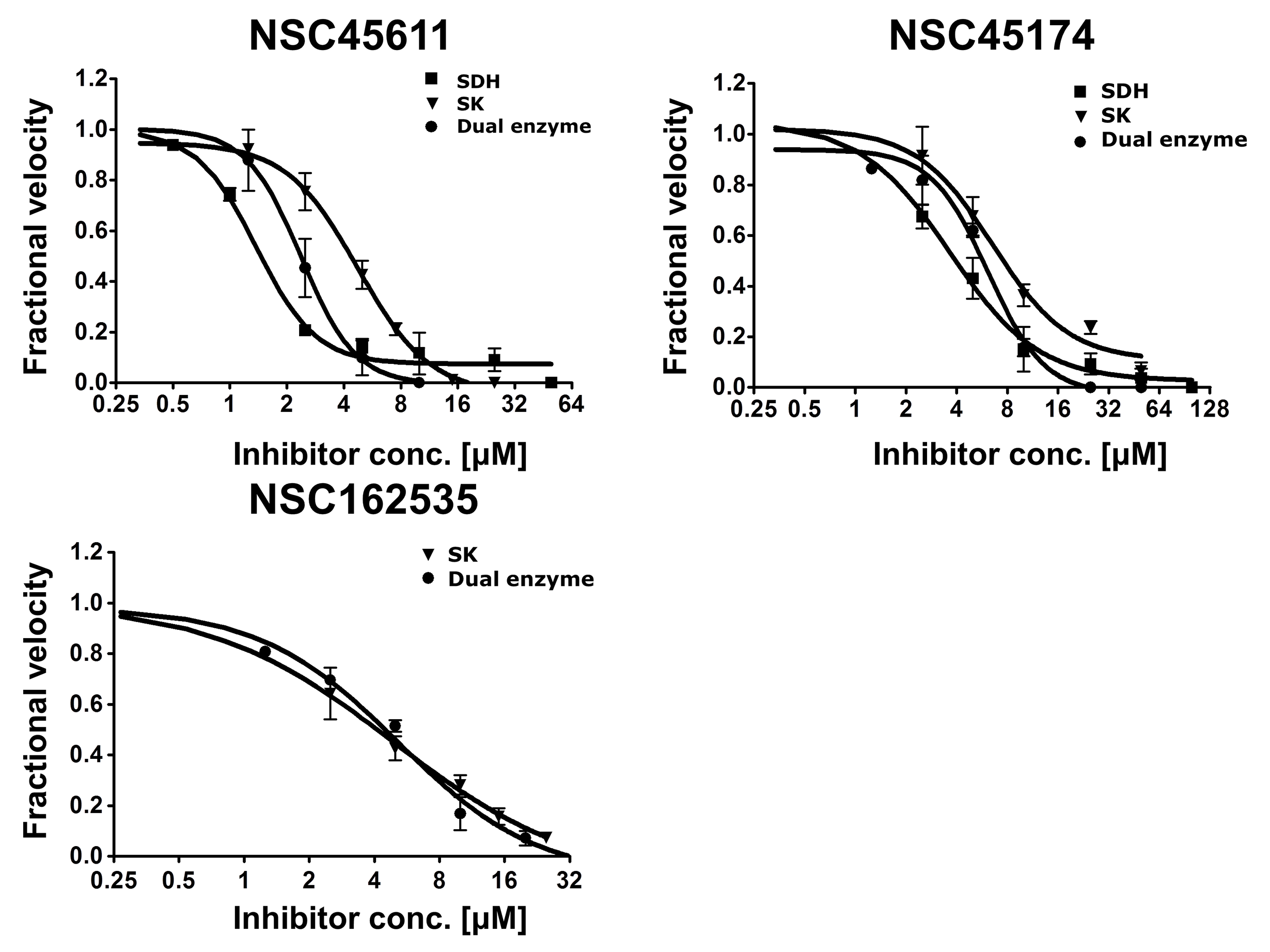

Supplement: Figure S4 — Comparison of three dose-response curves of shikimate dehydrogenase (SDH), shikimate kinase (SK), and dual enzyme (SDH and SK) activities on three compounds, NSC45611, NSC45174, and NSC162535 (specific for SK). For NSC45611 and NSC45174, at inhibitor concentrations greater than the IC90 value, the dual enzyme curves swiftly approached approximately 0, revealing the greater combined inhibitory effect. In contrast, there were nearly identical profiles for the SK-specific inhibitor (NSC162535). (TIF) [file pcbi.1003127.s004.tif]

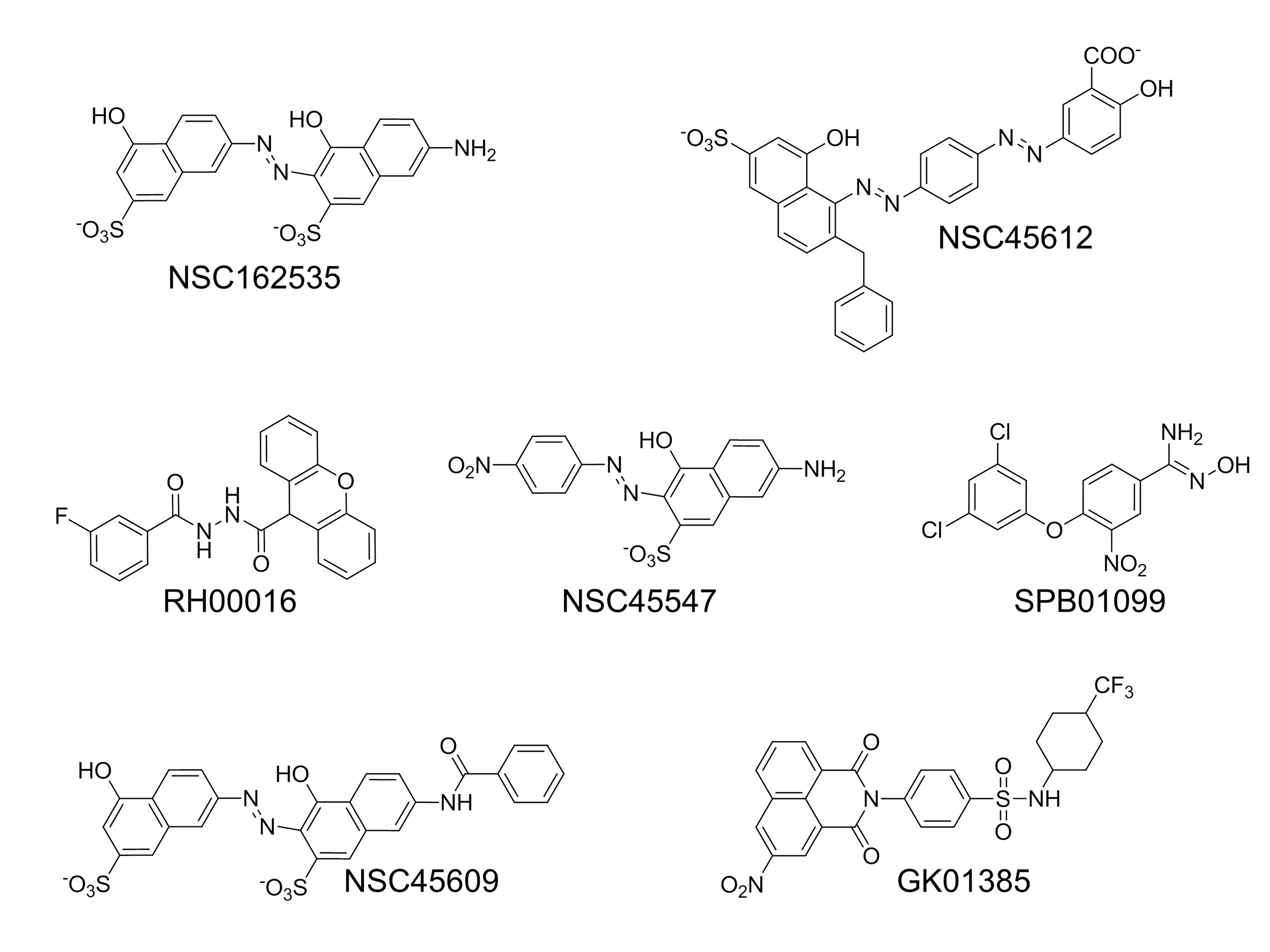

Supplement: Figure S5 — Inhibitors of shikimate kinase used for assessing performance. (TIF) [file pcbi.1003127.s005.tif]

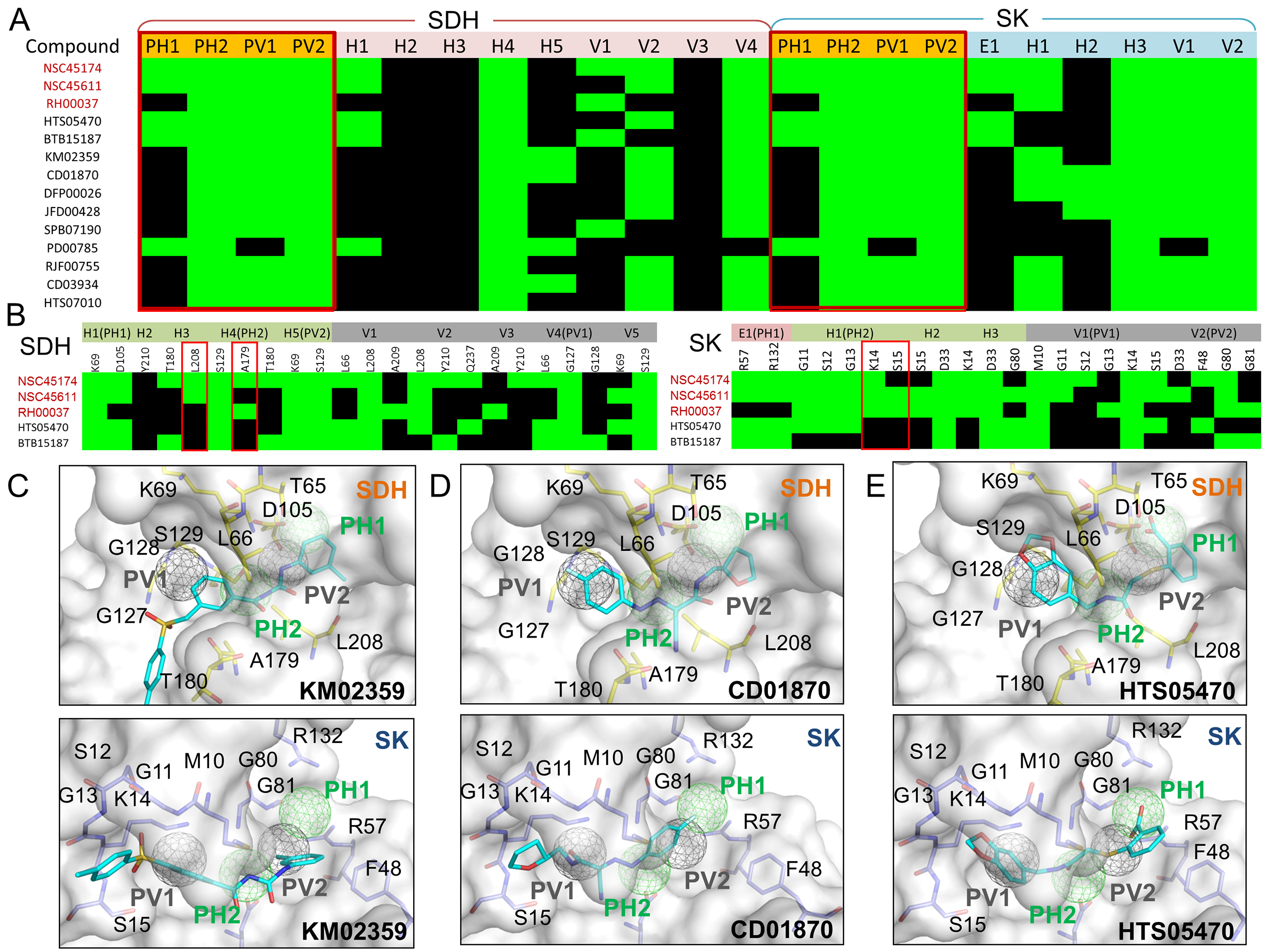

Supplement: Figure S6 — Interaction profile analyses of tested compounds. (A) Compound-anchor interaction profile of top-ranked compounds. (B) Compound-residue interaction profile of compounds matching the four pathway anchors. Docked poses of the top-ranked compounds (C) KM02359, (D) CD01870, and (E) HTS05470. (TIF) [file pcbi.1003127.s006.tif]

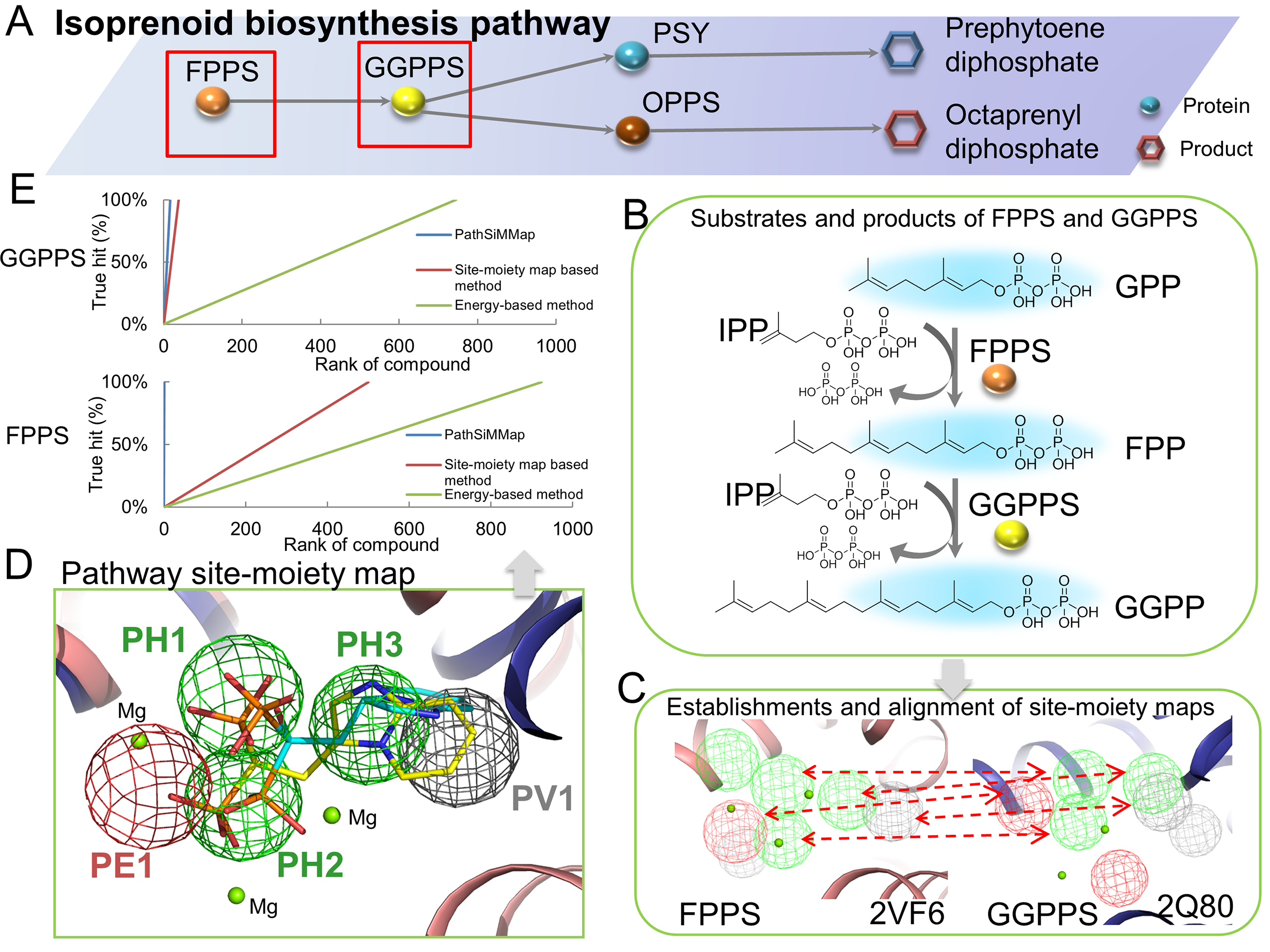

Supplement: Figure S7 — Pathway site-moiety maps of FPPS and GGPPS in isoprenoid biosynthesis pathway. (A) The proteins in the isoprenoid biosynthesis pathway. Among these protein, FPPS and GGPPS are directly connected. (B) Chemical reactions of FPPS and GGPPS. FPPS converts GPP to FPP using IPP, and then GGPPS converts FPP to GGPP along with IPP. The compounds (GPP, FPP, and GGPP) share the same substructure (blue region). (C) Establishment and alignment of site-moiety maps of FPPS and GGPPS. Electrostatic, hydrogen-bonding, and van der Waals anchors are colored in red, green, and gray, respectively. (D) Pathway anchors of FPPS and GGPPS. The multitarget inhibitor, minodronic acid, matched the PH1, PH2, PH3, and PV1 anchors. The docking poses in FPPS and GGPPS are represented by yellow and cyan, respectively. (E) Performance of the pathway-based screening strategy for the multitarget inhibitor compared with the site-moiety map-based and energy-based (GEMDOCK) methods. (TIF) [file pcbi.1003127.s007.tif]

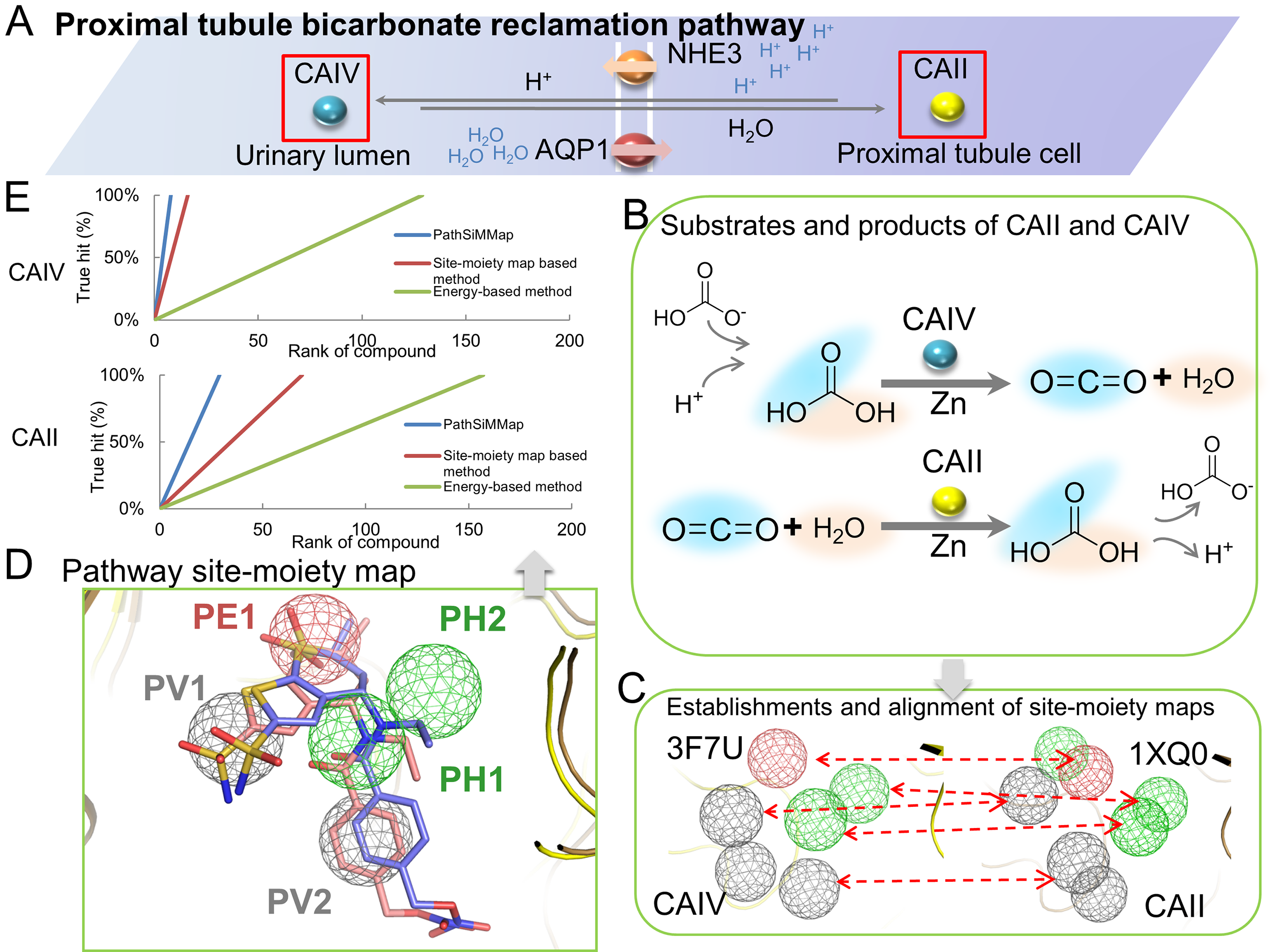

Supplement: Figure S8 — Pathway site-moiety maps of CAIV and CAII in proximal tubule bicarbonate reclamation pathway. (A) The proteins in the proximal tubule bicarbonate reclamation pathway. CAIV and CAII are directly connected. (B) Chemical reactions of CAIV and CAII. CAIV catalyzes HCO3 − and H+ into H2O and CO2. CAII then converts CO2 into HCO3 −. (C) Establishment and alignment of site-moiety maps of CAIV and CAII. Hydrogen-bonding and van der Waals anchors are colored in green and gray, respectively. (D) Pathway anchors of CAIV and CAII The multitarget inhibitor, NCX265, matched the PE1, PH1, PV1, and PV2 anchors. The docking poses of NCX265 in CAIV and CAII are represented by pink and purple, respectively. (E) Performance of the pathway-based screening strategy for the multitarget inhibitor compared with the site-moiety map-based and energy-based (GEMDOCK) methods. (TIF) [file pcbi.1003127.s008.tif]

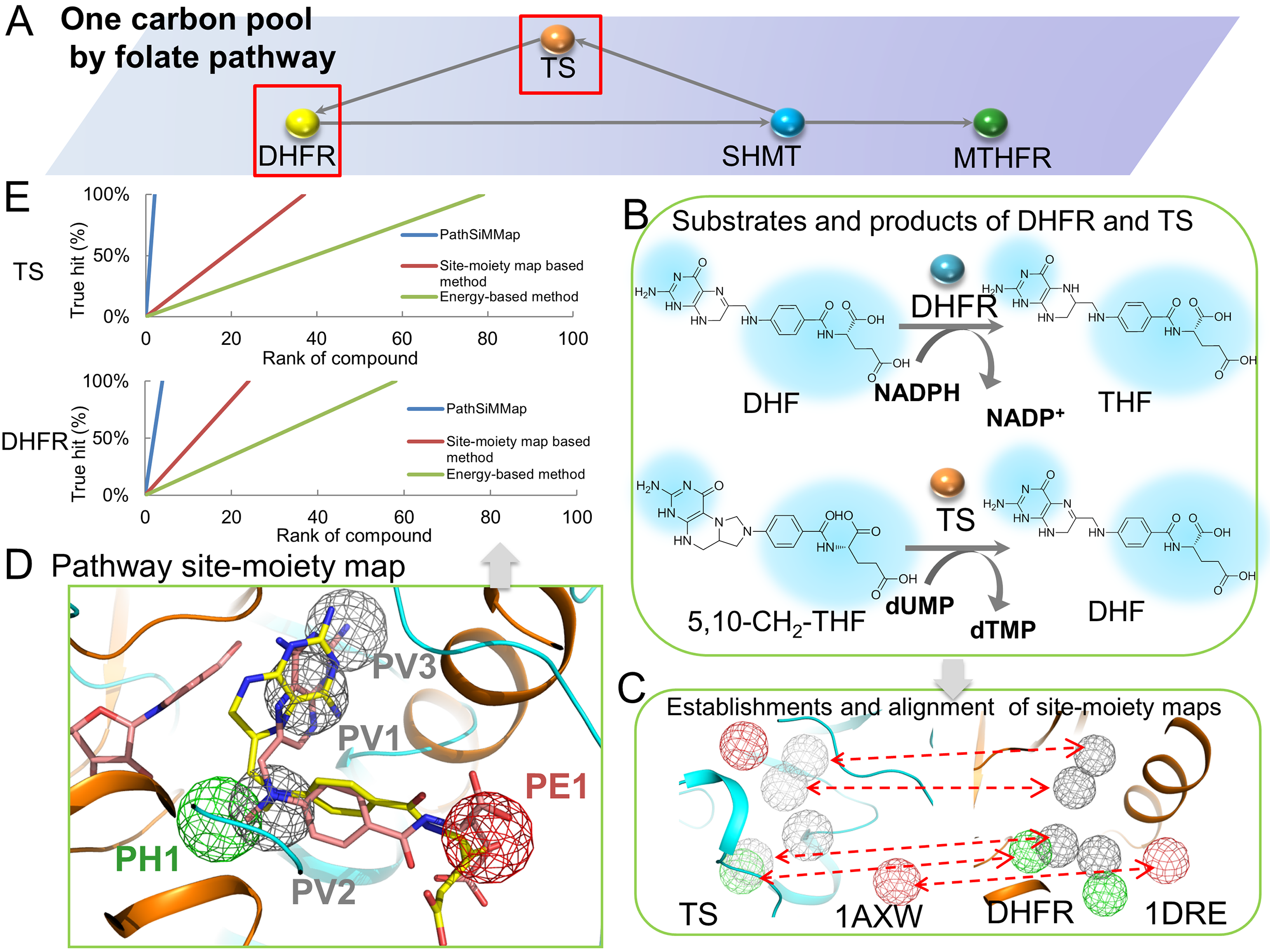

Supplement: Figure S9 — Pathway site-moiety maps of DHFR and TS in one carbon pool by folate pathway. (A) The proteins in the one carbon pool by folate pathway. Among these proteins, DHFR and TS are connected in the pathway. (B) Chemical reactions of DHFR and TS. DHFR catalyzes DHF to THF by NADPH. TS converts 5,10-CH2-THF to DHF using dUMP. These compounds share similar scaffolds. (C) Establishment and alignment of the site-moiety maps of DHFR and TS. Electrostatic, hydrogen-bonding, and van der Waals anchors are colored in red, green, and gray, respectively. (D) Pathway anchors of DHFR and TS. Methotrexate matched the PE1, PV1, PV2, and PV3 anchors. The docking poses of Methotrexate in DHFR and TS are represented by pink and yellow, respectively. (E) Performance of the pathway-based screening strategy for the multitarget inhibitor compared with the site-moiety map-based and energy-based (GEMDOCK) methods. (TIF) [file pcbi.1003127.s009.tif]

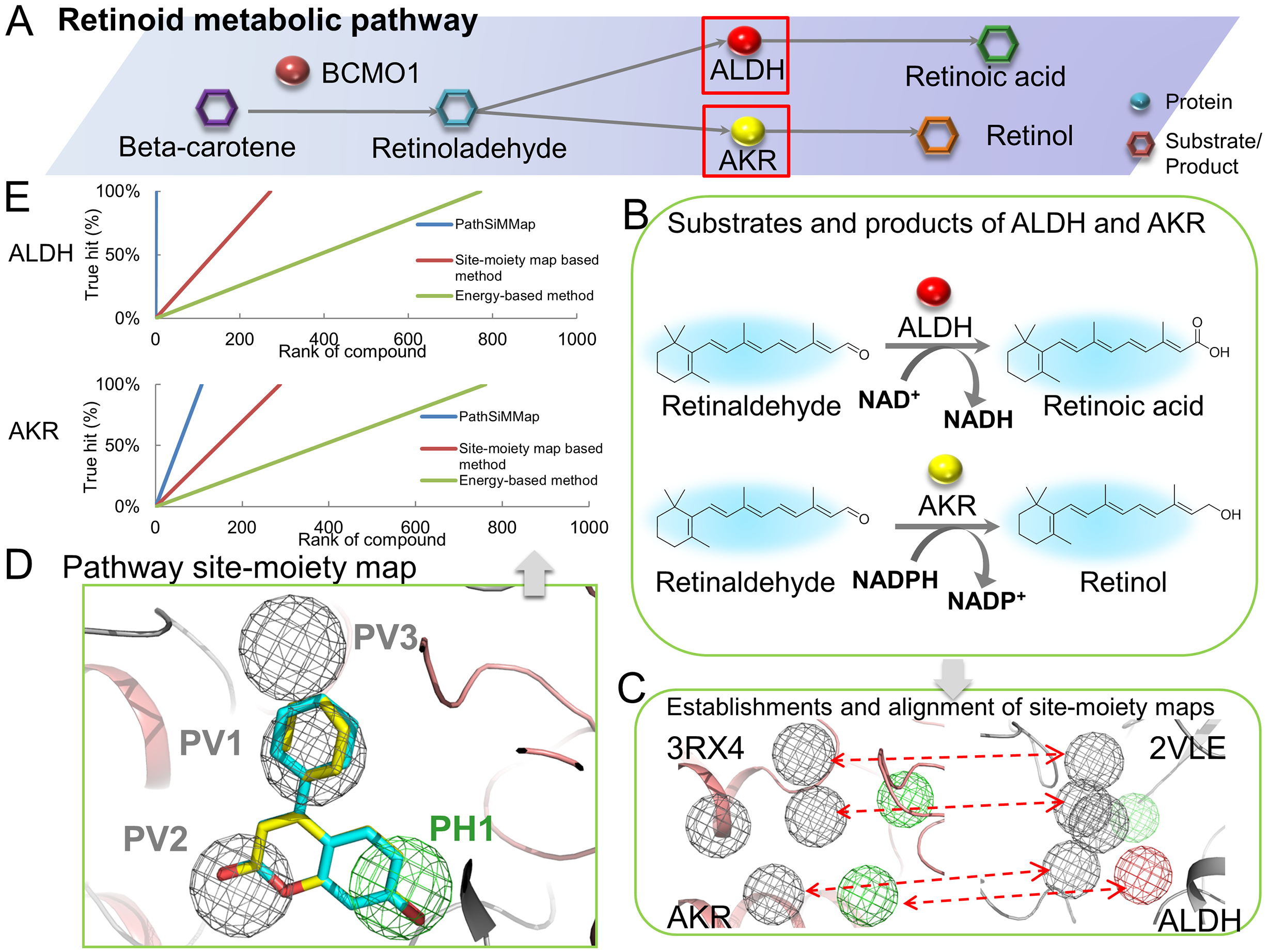

Supplement: Figure S10 — Pathway site-moiety maps of ALDH and AKR in retinoid metabolic pathway. (A) The proteins in the retinoid metabolic pathway. Among these proteins, ALDH and AKR are indirectly connected in the branched pathway. (B) Chemical reactions of ALDH and AKR. The former converts retinaldehyde into retinoic acid by NAD+. The latter catalyzes retinaldehyde into retinol using NADPH. (C) Establishment and alignment of the site-moiety maps of ALDH and AKR. Electrostatic, hydrogen-bonding, and van der Waals anchors are colored in red, green, and gray, respectively. (D) Pathway anchors of ALDH and AKR. Their common inhibitor, 7-hydroxy-4-phenylcoumarin, matched the PH1, PV1, and PV2 anchors. The docking poses of 7-hydroxy-4-phenylcoumarin in ALDH and AKR are represented by yellow and cyan, respectively. (E) Performance of the pathway-based screening strategy for the multitarget inhibitor compared with the site-moiety map-based and energy-based (GEMDOCK) methods. (TIF) [file pcbi.1003127.s010.tif]

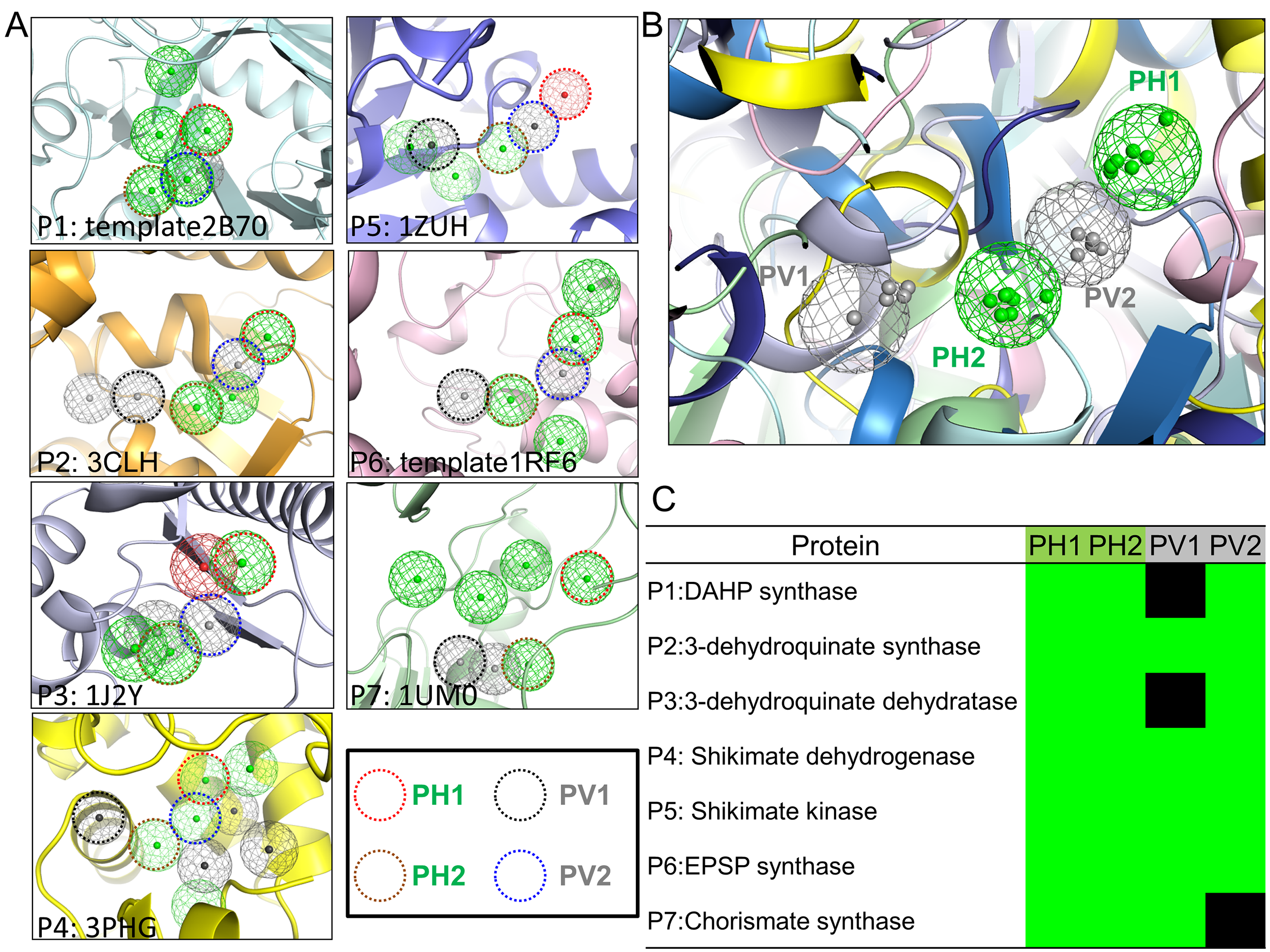

Supplement: Figure S11 — Anchor alignment for pathway anchors of seven proteins in shikimate pathway. (A) Site-moiety maps of DAHP synthase (template 2B70), 3-dehydroquinate synthase (3CLH), 3-dehydroquinate dehydratase (1J2Y), shikimate dehydrogenase (3PHG), shikimate kinase (1ZUH), EPSP synthase (template 1RF6), and chorismate synthase (1UM0). Negatively charged, hydrogen-bonding, and van der Waals anchors are colored in red, green, and gray, respectively. (B) Aligned anchors of the seven site-moiety maps (sphere) and the pathway anchor (mesh). (C) Anchor profile of the seven proteins. A cell is colored in green if the protein has the pathway anchor; otherwise the region is colored in black. (TIF) [file pcbi.1003127.s011.tif]

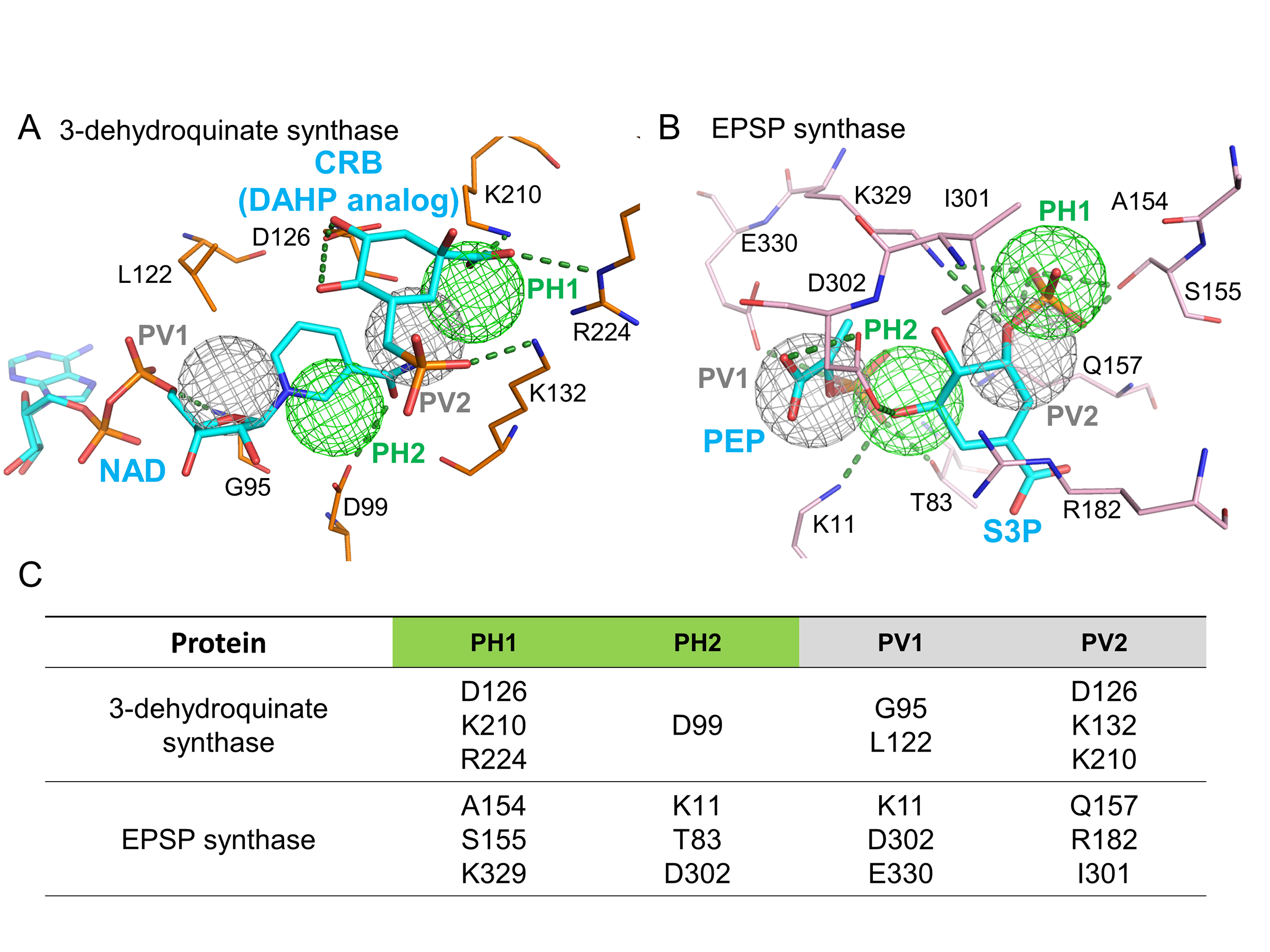

Supplement: Figure S12 — Pathway site-moiety map of 3-dehydroquinate synthase and EPSP synthase. Relationship between the pathway anchors and substrate/cofactor for (A) 3-dehydroquinate synthase and (B) EPSP synthase. The ligands of 3-dehydroquinate synthase are CRB (DAHP analog) (PDB code 1DQS, a 3-dehydroquinate synthase structure of Emericella nidulans) and NAD+. The EPSP synthase of Helicobacter pylori was modeled using a template structure (PDB code 1RF6). The ligands of EPSP synthase are shikimate-3-phosphate and PEP (PDB code 2O0E, an EPSP synthase structure of Mycobacterium tuberculosis). Hydrogen-bonding interactions between ligand and pathway anchor residues are represented as green dashes. (C) Pathway anchor residues of 3-dehydroquinate synthase and EPSP synthase. (TIF) [file pcbi.1003127.s012.tif]

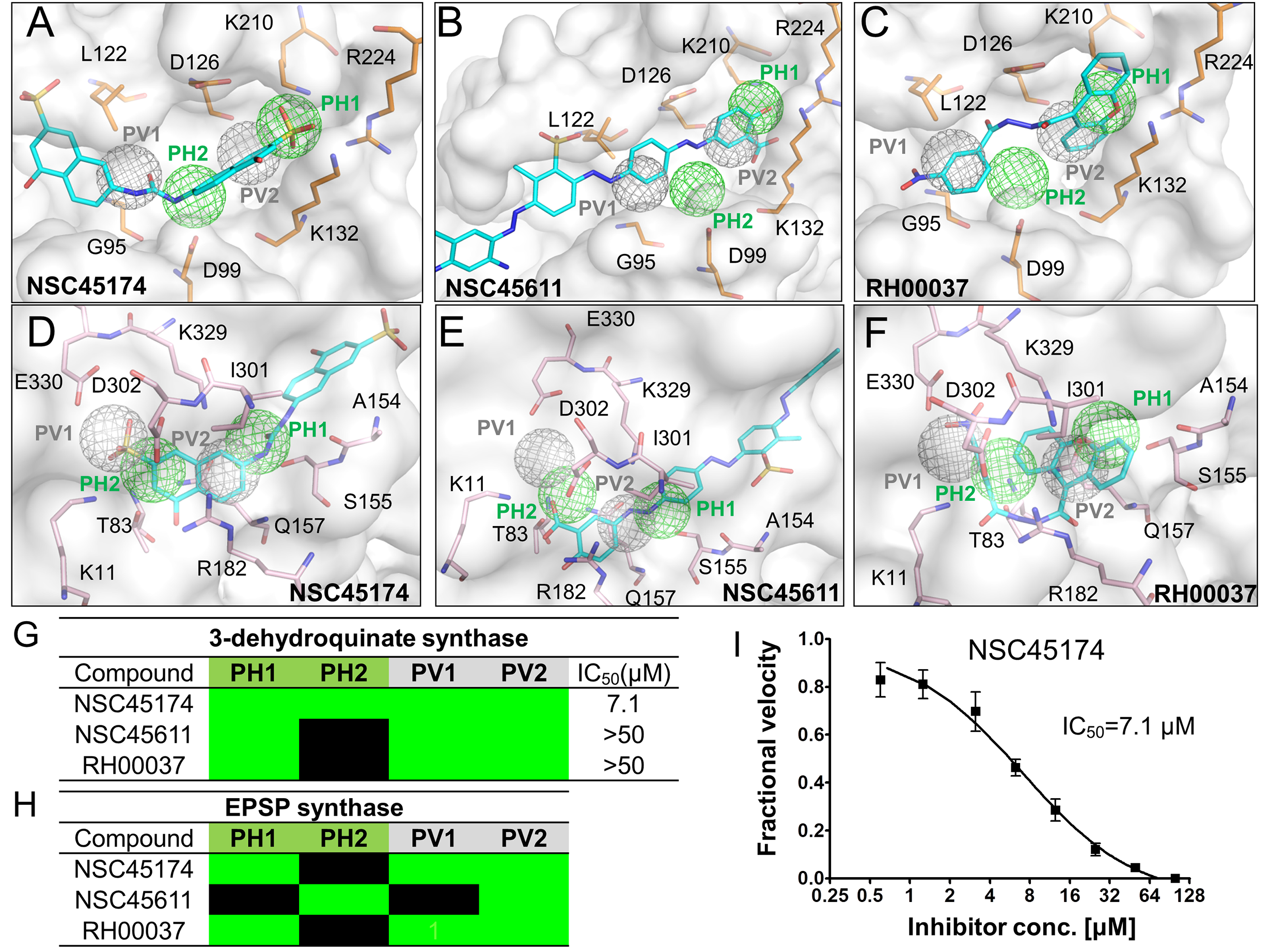

Supplement: Figure S13 — Relationship between pathway anchors and docking poses of NSC45174, NSC45611, and RH00037 for 3-dehydroquinate synthase and EPSP synthase. Docking poses of these compounds and anchor residues of (A–C) 3-dehydroquinate synthase and (D–F) EPSP synthase. Compound-pathway anchor profiles of (G) 3-dehydroquinate synthase and (H) EPSP synthase. A cell is colored in green if the compound matches the pathway anchor; otherwise the region is colored in black. (I) Dose-response curve of 3-dehydroquinate synthase activity on NSC45174. (TIF) [file pcbi.1003127.s013.tif]

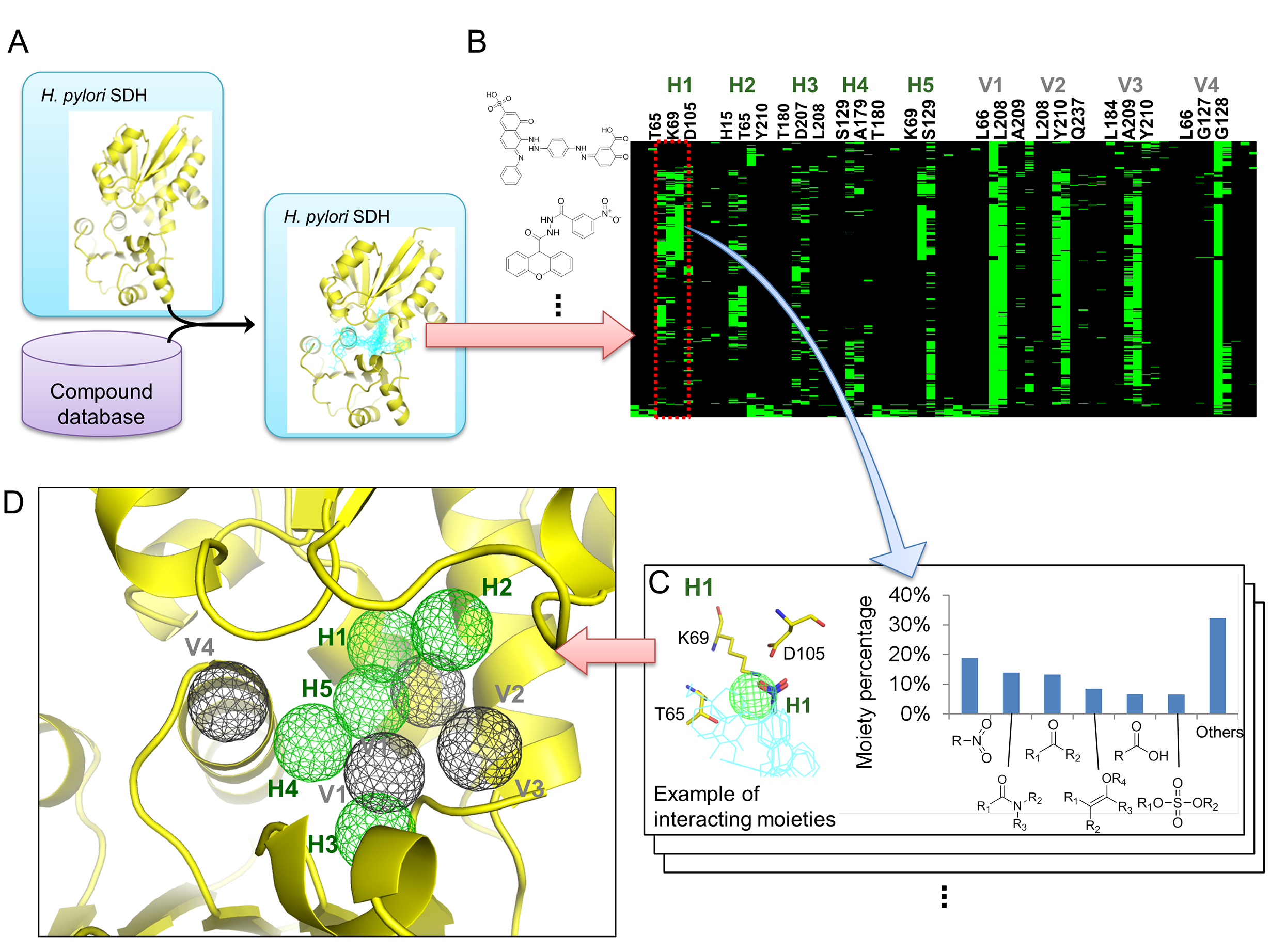

Supplement: Figure S14 — Establishment of a site-moiety map for a protein binding site using shikimate dehydrogenase. (A) Molecular docking for the screening target. (B) Merged protein–compound interaction profiles including electrostatic, hydrogen-bonding, and van der Waals profiles. A cell is colored in green if there is interaction (electrostatic, hydrogen-bonding, or van der Waals) between a compound moiety and a residue; otherwise the region is colored in black. (C) An anchor of the site-moiety map shown as an example. An anchor includes conserved interacting residues, moiety preferences, and interaction type. The example is hydrogen-bonding anchor, including a binding pocket consisting of polar residues, T65, K69, and D105. (D) Site-moiety map of SDH. The map consists of five hydrogen-bonding anchors (H1–H5), and four van der Waals anchors (V1–V4). Hydrogen-bonding and van der Waals anchors are colored in green and gray, respectively. (TIF) [file pcbi.1003127.s014.tif]

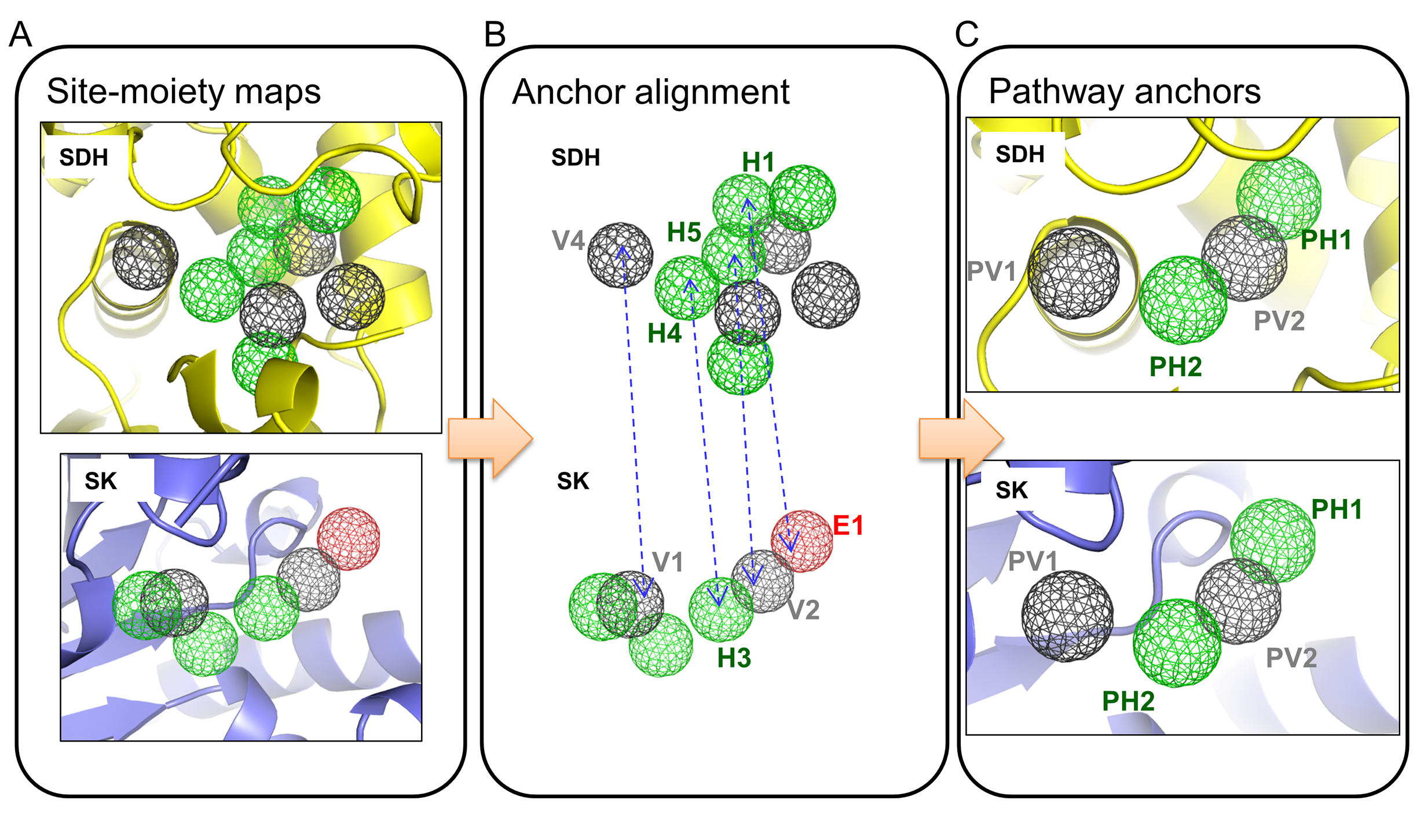

Supplement: Figure S15 — Identification of pathway anchors using the anchor-based alignment method. (A) Site-moiety maps of SDH and SK. (B) Alignment process and aligned anchors. SDH anchors V4, H4, H5, and H1 were aligned to SK anchors V1, H3, V2, and E1, respectively. (C) Pathway anchors of SDH and SK. (TIF) [file pcbi.1003127.s015.tif]
