# Supplementary material for: Pathway-based Screening Strategy for Multitarget Inhibitors of Diverse Proteins in Metabolic Pathways
Source: PLoS Comput Biol. 2013 Jul 4;9(7):e1003127. doi: 10.1371/journal.pcbi.1003127 (PMC3701698; doi:10.1371/journal.pcbi.1003127)
Supplement: Table S1 — Tested compound ranks of the SDH inhibitors. (DOC) [file pcbi.1003127.s016.doc]

**Table S1.** Tested compound ranks of the SDH inhibitors

| Compound | PathSiMMap rank (SDH+SK) | Site-moiety map rank (SDH) | GEMDOCK rank |
| --- | --- | --- | --- |
| NSC45174 | 13 | 177 | 3810 |
| NSC45611 | 80 | 879 | 4293 |
| HTS02876 | 79 | 185 | 5757 |
| NRB03174 | 93 | 246 | 2314 |
| RH00037 | 443 | 1592 | 2956 |
