# Supplementary material for: Pathway-based Screening Strategy for Multitarget Inhibitors of Diverse Proteins in Metabolic Pathways
Source: PLoS Comput Biol. 2013 Jul 4;9(7):e1003127. doi: 10.1371/journal.pcbi.1003127 (PMC3701698; doi:10.1371/journal.pcbi.1003127)
Supplement: Table S2 — Tested compound ranks of the SK inhibitors. (DOC) [file pcbi.1003127.s017.doc]

**Table S2.** Tested compound ranks of the SK inhibitors

| Compound | PathSiMMap rank (SDH+SK) | Site-moiety map rank (SK) | GEMDOCK rank |
| --- | --- | --- | --- |
| NSC45174 | 43 | 79 | 133 |
| NSC45611 | 61 | 260 | 1217 |
| RH00037 | 725 | 766 | 392 |
| NSC162535 | 26 | 26 | 2438 |
| NSC45612 | 35 | 48 | 39 |
| NSC45547 | 103 | 636 | 5833 |
| GK01385 | 433 | 389 | 2440 |
| SPB01099 | 644 | 622 | 5533 |
| NSC45609 | 1416 | 2062 | 5259 |
| RH00016 | 1527 | 2219 | 5995 |
