# Supplementary material for: Pathway-based Screening Strategy for Multitarget Inhibitors of Diverse Proteins in Metabolic Pathways
Source: PLoS Comput Biol. 2013 Jul 4;9(7):e1003127. doi: 10.1371/journal.pcbi.1003127 (PMC3701698; doi:10.1371/journal.pcbi.1003127)
Supplement: Table S3 — PathSiMMap ranks and IC50 values of the tested multitarget compound candidates. (DOC) [file pcbi.1003127.s018.doc]

**Table S3.** PathSiMMap ranks and IC_50_ values of the tested multitarget compound candidates

| Compound ID | Compound structure | Consensus rank | SDH PathSiMMap rank | SDH IC_50_ (μM) | SK PathSiMMap rank | SK IC_50_ (μM) |
| --- | --- | --- | --- | --- | --- | --- |
| NSC45174 |  | 3 | 13 | 3.6 | 43 | 7.8 |
| NSC45611 |  | 7 | 80 | 1.4 | 61 | 4.8 |
| RH00037 |  | 34 | 443 | 24.8 | 725 | 23.8 |
| BTB15187 |  | 9 | 67 | -^a^ | 105 | - |
| HTS05470 |  | 10 | 74 | - | 106 | - |
| KM02359 |  | 26 | 191 | - | 666 | - |
| CD01870 |  | 12 | 148 | - | 107 | - |
| DFP00026 |  | 15 | 319 | - | 104 | - |
| JFD00428 |  | 44 | 609 | - | 669 | - |
| SPB07190 |  | 86 | 534 | - | 1537 | - |
| PD00785 |  | 132 | 620 | - | 2387 | - |
| RJF00755 |  | 36 | 521 | - | 668 | - |
| CD03934 |  | 23 | 134 | - | 667 | - |
| HTS07010 |  | 47 | 623 | - | 665 | - |

^a^ Inhibition percentage < 50% at 100 μM.
