# Supplementary material for: Pathway-based Screening Strategy for Multitarget Inhibitors of Diverse Proteins in Metabolic Pathways
Source: PLoS Comput Biol. 2013 Jul 4;9(7):e1003127. doi: 10.1371/journal.pcbi.1003127 (PMC3701698; doi:10.1371/journal.pcbi.1003127)
Supplement: Table S4 — PathSiMMap ranks and IC50 values of the tested SDH-specific compound candidates. (DOC) [file pcbi.1003127.s019.doc]

**Table S4.** PathSiMMap ranks and IC_50_ values of the tested SDH-specific compound candidates

| Compound ID | Compound structure | SDH PathSiMMap rank | SDH IC_50_ (μM) | SK IC_50_ (μM) |
| --- | --- | --- | --- | --- |
| NRB03174 |  | 93 | 9.7 | -^a^ |
| HTS02873 |  | 79 | 4.9 | - |
| HTS04060 |  | 149 | - | - |
| HTS08853 |  | 249 | - | - |
| DFP00083 |  | 316 | - | - |
| NSC71881 |  | 373 | - | - |

^a^ Inhibition percentage < 50% at 100 μM.
