# Supplementary material for: Pathway-based Screening Strategy for Multitarget Inhibitors of Diverse Proteins in Metabolic Pathways
Source: PLoS Comput Biol. 2013 Jul 4;9(7):e1003127. doi: 10.1371/journal.pcbi.1003127 (PMC3701698; doi:10.1371/journal.pcbi.1003127)
Supplement: Table S5 — Selected pathways, proteins, and multitarget inhibitors used for verifying the pathway-based screening strategy. (DOC) [file pcbi.1003127.s020.doc]

**Table S5.** Selected pathways, proteins, and multitarget inhibitors used for verifying the pathway-based screening strategy

| Pathway | Selected proteins | Group | Multitarget inhibitor |
| --- | --- | --- | --- |
| Isoprenoid biosynthesis pathway | GGPPS (2Q80), FPS (2VF6) | Directly connected | Minodronic acid |
| Proximal tubule bicarbonate reclamation | CAⅡ(1XQ0), CAⅣ(3F7U) | Directly connected | NCX265 |
| One carbon pool by folate pathway | DHFR (1DRE), TS (1AXW) | Directly connected | Methotrexate |
| Retinoid metabolic pathway | ALDH (2VLE), AKR (3RX4) | Indirectly connected | 7-hydroxy-4-phenylcoumarin |
