# Supplementary material for: Pathway-based Screening Strategy for Multitarget Inhibitors of Diverse Proteins in Metabolic Pathways
Source: PLoS Comput Biol. 2013 Jul 4;9(7):e1003127. doi: 10.1371/journal.pcbi.1003127 (PMC3701698; doi:10.1371/journal.pcbi.1003127)
Supplement: Text S1 — Application of pathway-based screening strategy on four pathways. (DOC) [file pcbi.1003127.s021.doc]

**Text S1. Application of pathway-based screening strategy on four pathways**

We selected four pathways to evaluate the pathway-based screening strategy for identifying multitarget inhibitors (Table S5) based on the following criteria: (1) The proteins, locating in a linear or branched pathway, perform a series of catalytic reactions, (2) These proteins can be inhibited by the same inhibitor with IC_50_ values <10 μM, and (3) The structures of these proteins are available. These proteins and their inhibitors were used to assess the performances of different strategies.

These four pathways can be divided into two groups based on the target protein locations in the pathways: (1) directly connected proteins, and (2) indirectly connected proteins. The first group includes the isoprenoid biosynthesis pathway, the proximal tubule bicarbonate reclamation pathway, and the one carbon pool by folate pathway, while the second group consists of the retinoid metabolic pathway. The isoprenoid biosynthesis pathway produces cholesterol and a number of non-sterol isoprenoids (Fig. S7). For this pathway, we selected directly connected FPPS and GGPPS as the target proteins. FPPS first converts GPP into FPP along with IPP, and then GGPPS converts FPP into GGPP using IPP . These metabolites share a common scaffold (blue part in Fig. S7B). Minodronic acid is reportedly a multitarget inhibitor for FPPS and GGPPS (Table S5) . The proximal tubule bicarbonate reclamation regulates blood pH by reabsorbing and generating bicarbonate (HCO_3_^−^) . CAII and CAIV are directly connected proteins in this pathway (Fig. S8) and can be inhibited by a multitarget inhibitor, NCX265 (Table S5). During this regulation process, HCO_3_^−^ and H^+^ are catalyzed into H_2_O and CO_2_ by CAIV (Fig. S8B). Next, CO_2_ diffuses into tubular cells and is converted into HCO_3_^−^ by CAII . The substrates/products of CAII and CAIV share similar substructures (blue and red parts in Fig. S8B).

The one carbon pool by folate pathway regulates the production of cofactors and vitamins (Fig. S9). Among these proteins in this pathway, connected DHFR and TS were selected as targets. DHFR converts DHF to THF via NADPH. TS converts 5,10-CH_2_-THF to DHF using dUMP (Fig. S9B). These metabolites share similar scaffolds. Methotrexate has been shown to simultaneously inhibit DHFR and TS (Table S5). The retinoid metabolic pathway plays an important role in many physiological processes, including embryonic development, differentiation, vision, and maintaining various types of epithelial maintenance cells (Fig. S10). ALDH and AKR are indirectly connected in this pathway. The former converts retinaldehyde to retinoic acid via NAD^+^ and the latter catalyzes retinaldehyde into retinol using NADPH. Their common inhibitor is 7-hydroxy-4-phenylcoumarin (Table S5).

The pathway-based screening strategy to discover multitarget inhibitors relies on the following criteria: (1) the proteins catalyze ligands with common substructures, and (2) these proteins share conserved binding environments and comparable anchors in their site-moiety maps. First, the site-moiety maps of the target proteins were established (Figs. S7C, S8C, S9C, and S10C). The pathway site-moiety maps of each pathway were then constructed by the anchor-based alignment method (Figs. S7D, S8D, S9D, and S10D). The pathway site-moiety maps of these four pathways and the shikimate pathway generally share 4–5 pathway anchors. For each pathway, we collected a compound set that included these multitarget inhibitors (Table S5) and 990 compounds randomly selected from the Available Chemical Directory proposed by Bissantz *et. al.*  to verify our pathway-based screening strategy. The PathSiMMap score was then applied to identify multitarget inhibitors from the screening compounds (multitarget inhibitors and 990 random compounds). The true-hit rates showed that the PathSiMMaps yielded better performance than the site-moiety maps and the energy-based methods (using GEMDOCK score) for identifying multitarget inhibitors (Figs. S7E, S8E, S9E, and S10E). These experimental results suggest that the pathway-based screening strategy can be successfully applied to these pathways for identifying multitarget inhibitors.

**References**

1. Kajiwara S, Fraser PD, Kondo K, Misawa N (1997) Expression of an exogenous isopentenyl diphosphate isomerase gene enhances isoprenoid biosynthesis in Escherichia coli. Biochemical Journal 324: 421-426.

2. Guo RT, Cao R, Liang PH, Ko TP, Chang TH, et al. (2007) Bisphosphonates target multiple sites in both cis- and trans-prenyltransferases. Proceedings of the National Academy of Sciences of the United States of America 104: 10022-10027.

3. Mao JH, Mukherjee S, Zhang Y, Cao R, Sanders JM, et al. (2006) Solid-state NMR, crystallographic, and computational investigation of Bisphosphonates and farnesyl diphosphate synthase-bisphosphonate complexes. Journal of the American Chemical Society 128: 14485-14497.

4. Boron WF (2006) Acid-base transport by the renal proximal tubule. Journal of the American Society of Nephrology 17: 2368-2382.

5. Steele RM, Benedini F, Biondi S, Borghi V, Carzaniga L, et al. (2009) Nitric oxide-donating carbonic anhydrase inhibitors for the treatment of open-angle glaucoma. Bioorganic & Medicinal Chemistry Letters 19: 6565-6570.

6. Ifergan I, Assaraf YG (2008) Molecular Mechanisms of Adaptation to Folate Deficiency. Folic Acid and Folates 79: 99-143.

7. Chu E, Drake JC, Boarman D, Baram J, Allegra CJ (1990) Mechanism of Thymidylate Synthase Inhibition by Methotrexate in Human Neoplastic Cell-Lines and Normal Human Myeloid Progenitor Cells. Journal of Biological Chemistry 265: 8470-8478.

8. Rajagopalan PTR, Zhang ZQ, McCourt L, Dwyer M, Benkovic SJ, et al. (2002) Interaction of dihydrofolate reductase with methotrexate: Ensemble and single-molecule kinetics. Proceedings of the National Academy of Sciences of the United States of America 99: 13481-13486.

9. Ruiz FX, Porte S, Pares X, Farres J (2012) Biological role of aldo-keto reductases in retinoic Acid biosynthesis and signaling. Front Pharmacol 3: 58.

10. Gao GY, Li DJ, Keung WM (2001) Synthesis of potential antidipsotropic isoflavones: Inhibitors of the mitochondrial monoamine oxidase-aldehyde dehydrogenase pathway. Journal of Medicinal Chemistry 44: 3320-3328.

11. Kato A, Kobayashi K, Narukawa K, Minoshima Y, Adachi I, et al. (2010) 6,7-Dihydroxy-4-phenylcoumarin as inhibitor of aldose reductase 2. Bioorganic & Medicinal Chemistry Letters 20: 5630-5633.

12. Bissantz C, Folkers G, Rognan D (2000) Protein-based virtual screening of chemical databases. 1. Evaluation of different docking/scoring combinations. J Med Chem 43: 4759-4767.
